# Mutant ANP induces mitochondrial and ion channel remodeling in a human iPSC–derived atrial fibrillation model

**DOI:** 10.1172/jci.insight.155640

**Published:** 2022-04-08

**Authors:** Olivia T. Ly, Hanna Chen, Grace E. Brown, Liang Hong, Xinge Wang, Yong Duk Han, Mahmud Arif Pavel, Arvind Sridhar, Mark Maienschein-Cline, Brandon Chalazan, Sang-Ging Ong, Khaled Abdelhady, Malek Massad, Lona Ernst Rizkallah, Jalees Rehman, Salman R. Khetani, Dawood Darbar

**Affiliations:** 1 Division of Cardiology, Department of Medicine;; 2 Department of Biomedical Engineering,; 3 Department of Physiology;; 4 Research Informatics Core, Research Resources Center;; 5 Department of Pharmacology and Regenerative Medicine; and; 6 Division of Cardiothoracic Surgery, Department of Surgery, University of Illinois at Chicago, Chicago, Illinois, USA.

**Keywords:** Cardiology, Genetics, Arrhythmias, Genetic diseases, iPS cells

## Abstract

Human induced pluripotent stem cell–derived cardiomyocytes (iPSC-CMs) can model heritable arrhythmias to personalize therapies for individual patients. Although atrial fibrillation (AF) is a leading cause of cardiovascular morbidity and mortality, current platforms to generate iPSC-atrial (a) CMs are inadequate for modeling AF. We applied a combinatorial engineering approach, which integrated multiple physiological cues, including metabolic conditioning and electrical stimulation, to generate mature iPSC-aCMs. Using the patient’s own atrial tissue as a gold standard benchmark, we assessed the electrophysiological, structural, metabolic, and molecular maturation of iPSC-aCMs. Unbiased transcriptomic analysis and inference from gene regulatory networks identified key gene expression pathways and transcription factors mediating atrial development and maturation. Only mature iPSC-aCMs generated from patients with heritable AF carrying the non-ion channel gene (*NPPA*) mutation showed enhanced expression and function of a cardiac potassium channel and revealed mitochondrial electron transport chain dysfunction. Collectively, we propose that ion channel remodeling in conjunction with metabolic defects created an electrophysiological substrate for AF. Overall, our electro-metabolic approach generated mature human iPSC-aCMs that unmasked the underlying mechanism of the first non-ion channel gene, *NPPA*, that causes AF. Our maturation approach will allow for the investigation of the molecular underpinnings of heritable AF and the development of personalized therapies.

## Graphical abstract

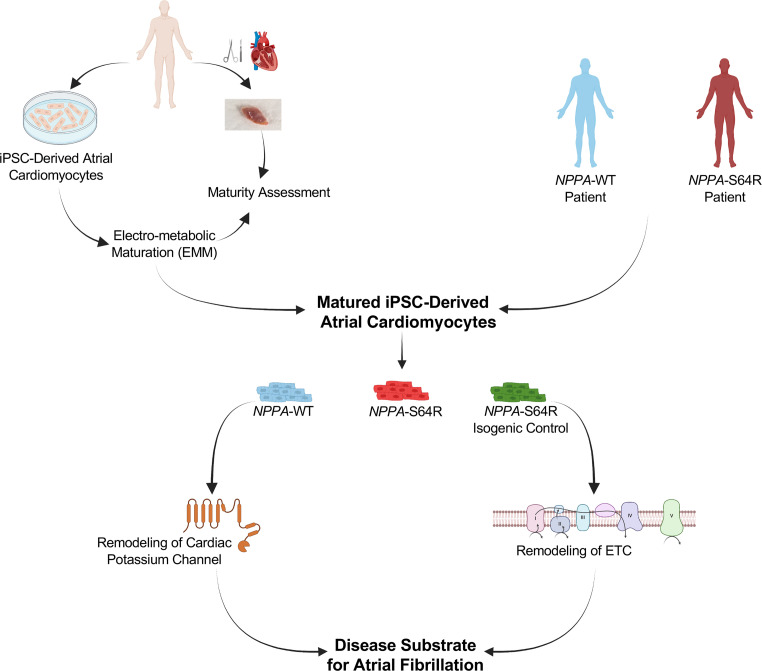

## Introduction

Atrial fibrillation (AF) affects over 33 million people worldwide and is associated with significant morbidity and mortality. Despite recent advances in catheter-based treatments, antiarrhythmic drugs remain an important treatment of AF, but their highly variable efficacies is concerning because membrane-active drugs are also associated with serious toxicities (1–3). The limited success of pharmacological therapy is due in part to poor understanding of the myocardial substrate for AF as human atrial tissue (HAT) is rarely available and existing in vitro and in vivo models carry several limitations. Tremendous progress has been made in defining the genetic basis of AF. Genome-wide association studies have identified over 100 AF loci (4), and family-based studies have implicated mutations in genes encoding ion channels, signaling molecules, and sarcomeric proteins (5–13). A gain-of-function mutation in *KCNQ1*, encoding the α subunit of the cardiac delayed slow rectifier potassium channel current (I_Ks_), shortened the atrial action potential duration (APD) and reduced the effective refractory period, thereby creating a reentrant substrate for familial AF (5). Such an understanding of the electrophysiological (EP) mechanism for specific familial AF mutations enables the development of targeted pharmacological therapy in patients carrying the disease-inducing mutations.

Human induced pluripotent stem cell–derived atrial cardiomyocytes (iPSC-aCMs) possess the complex array of ion channels that make up the atrial AP and hold great promise for modeling AF if they can be induced to more faithfully reproduce characteristics of human atria (14, 15). Modeling patient-specific mutations associated with AF using mature iPSC-aCMs offers a powerful, naturally integrated system with distinct advantages over heterologous expression and animal models (16). Heterologous expression in noncardiac cells is suitable for selected molecular studies but only permits the investigation of individual ion channels, while iPSC-aCMs can also demonstrate the interactions between ion channel function and other genes or proteins that promote AF. Importantly, murine models do not express several key genes/proteins, particularly potassium channels, crucial to generating the human atrial AP. However, the immaturity of iPSC-aCMs limits their fidelity to model AF phenotypes in vitro. Enhancing iPSC-aCM maturity is important for elucidating the underlying cellular mechanisms of AF and the identification of developmental pathways for therapeutic targeting. Studies in ventricular iPSC-CMs have shown that certain hormones and postnatal factors promote the maturation of calcium handling, structural gene, and metabolic machinery (17–20). Furthermore, lipid cocktails induce metabolic changes, and electrical stimulation (ES) improves cellular structure, intercellular communication, and EP in ventricular iPSC-CMs (21–24). Nonetheless, differentiation of iPSC-aCMs have been limited with immature phenotypes, and studies have failed to recapitulate the impact of atrial-selective potassium currents like I_Ks_ on repolarization (25–27). We applied an electro-metabolic maturation (EMM) approach of biochemical stimulation with a) hormones known to promote cardiac maturation and function — triiodothyronine (T), insulin-like growth factor-1 (I), and dexamethasone (D) (known as TID); b) bioenergetic conditioning with fatty acid (FA) supplementation as the primary source of energy in the adult heart (28); and c) ES to synergistically promote iPSC-aCM maturity. Using the patient’s own atrial tissue as a benchmark, we assessed the EP, structural, metabolic, and molecular maturation of iPSC-aCMs.

The non-ion channel gene *NPPA* encoding atrial natriuretic peptide (ANP) is the first non-ion channel gene linked to familial AF (10). However, the cellular mechanisms by which mutations in this gene cause AF remain unclear because iPSC-aCMs are immature and existing murine models are mechanistically limited. We hypothesized that the EMM approach would unmask the underlying cellular mechanisms of heritable AF associated with an *NPPA* mutation. To test this hypothesis, we generated iPSCs from patients with heritable AF carrying the *NPPA*-S64R mutation (29) and showed that mature iPSC-aCMs enhanced the expression and function of I_Ks_ and revealed downregulation of mitochondrial electron transport (ETC) chain activity when compared with immature aCMs, creating an EP substrate for AF. Our EMM approach may be used to further elucidate molecular mechanisms underlying AF and to test personalized therapies.

## Results

**Figure 1:**
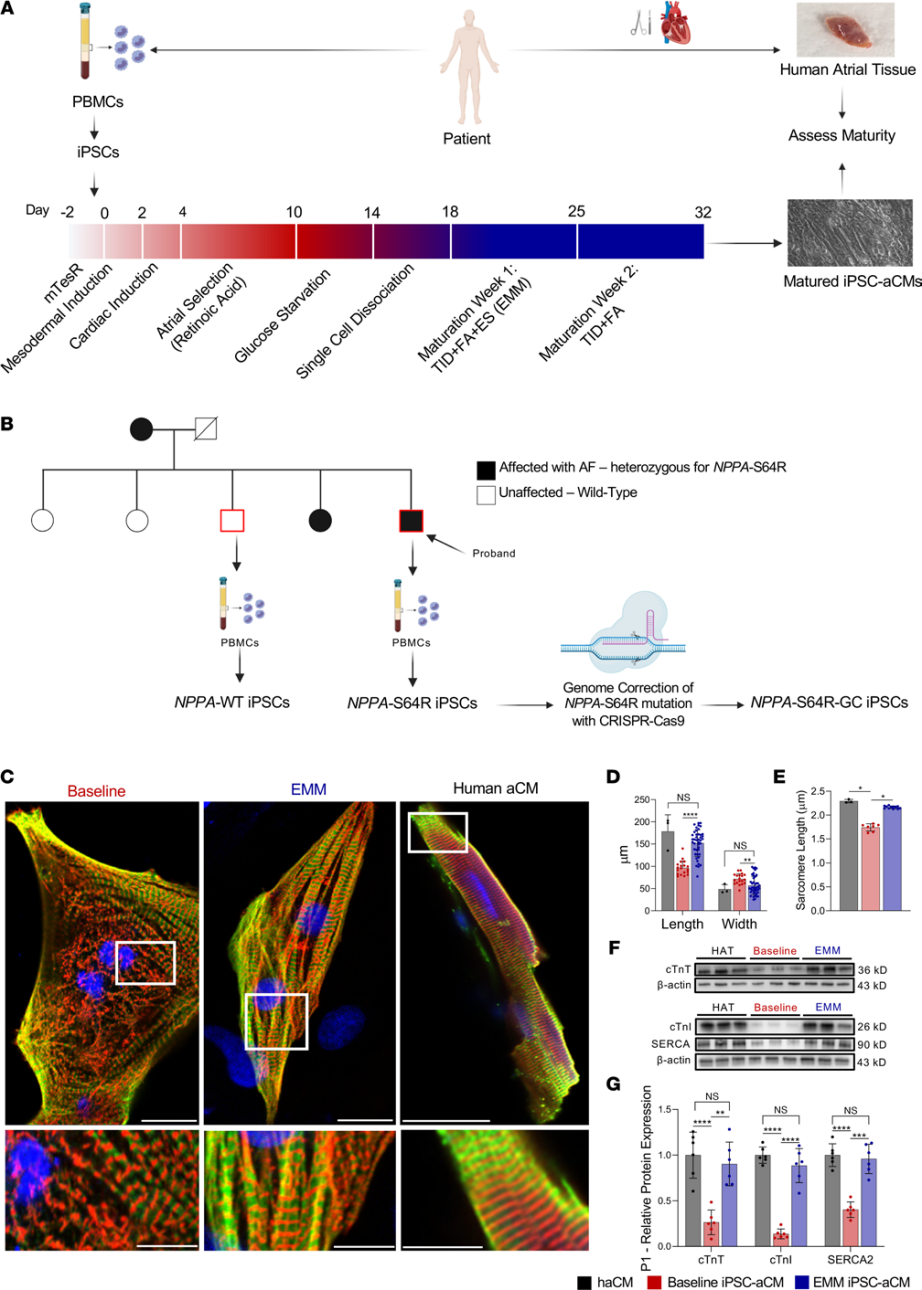
Overall study design. (**A**) Maturation methodology and timeline. Both patient and human atrial tissue (HAT) were obtained from a patient. The peripheral blood mononuclear cells (PBMCs) were reprogrammed into iPSCs, differentiated into induced pluripotent stem cell–derived atrial cardiomyocytes (iPSC-aCMs), then matured with 2 weeks of EMM. (Created using BioRender.) (**B**) Generation of *NPPA*-wild-type (WT), *NPPA*-S64R, and *NPPA*-S64R-genome-corrected (GC) iPSC lines from the atrial fibrillation (AF) kindred carrying the *NPPA*-S64R mutation. PBMCs were obtained from II-3 (*NPPA*-WT) and II-5 (*NPPA*-S64R; proband) and reprogrammed into iPSCs. *NPPA*-S64R-GC was created using CRISPR/Cas9 to genome correct the S64R to the WT sequence using iPSCs from II-5. (Created using BioRender.) (**C**–**G**) EMM enhanced the structural maturity of human iPSC-aCMs. (**C**) Immunofluorescence staining for cardiac troponin T (cTnT) and α-actinin showed that EMM resulted in a more elongated cell morphology and sarcomeric organization in both the periphery and perinuclear region (middle) compared with baseline iPSC-aCMs (left). This improvement in structural organization is similar to that of human aCMs (right). Scale bars for full image represent 30 μm. Scale bars for inset represent 15 μm. (**D** and **E**) EMM iPSC-aCMs demonstrated increased length and decreased width (2-way ANOVA with Bonferroni’s correction) (**D**), as well as increased sarcomeric length (1-way ANOVA with Bonferroni’s correction) (**E**). (**F** and **G**) Western blot analysis of EMM iPSC-aCMs also demonstrated increased protein expression of cTnT, cTnI, and sarco/endoplasmic reticulum calcium-ATPase (SERCA2) to a level no longer significantly different from HAT; (*n* = 6); (2-way ANOVA with Bonferroni’s correction). (Note: In order to probe for cTnI and SERCA2, we ran the protein lysates on a single SDS-PAGE gel, and transferred the gel onto a single PVDF membrane. The membrane was then cut to individually probe for SERCA2, cTnI, and β-actin, hence why β-actin is the same for SERCA2 and cTnI.) *P < 0.05, **P < 0.01, ***P < 0.001, ****P < 0.0001.

We differentiated human iPSC-aCMs from 3 independently derived iPSC lines, and 3 independent clones from each cell line, with each exhibiting normal pluripotency profile and karyotype, with the iPSC-CMs selected for an atrial subtype with retinoic acid treatment for 5 days (Figure 1A). Immunofluorescence (IF) staining for the atrium-specific marker K_v_1.5, real-time quantitative polymerase chain reaction (RT-PCR), and flow cytometry comparing ventricular and atrial markers demonstrated the atrial phenotype of the iPSC-aCMs (16, 30). P1 and P2 lines were generated from patients with no prior diagnosis of AF recruited to the Human Cardiac Atrial Tissue Biorepository, with the third derived from a control (L3) (Supplemental Table 1; supplemental material available online with this article; https://doi.org/10.1172/jci.insight.155640DS1) (31). We obtained HAT at the time of cardiac surgery from P1 and P2 for a comprehensive comparison of iPSC-aCM maturity with the patient’s own aCMs (Figure 1A). The P1, P2, and L3 lines, generated from individuals with no history of AF who did not carry the *NPPA*-S64R mutation, were control lines used to optimize the EMM protocol. We generated iPSC-aCMs from a family carrying an *NPPA*-S64R mutation and an isogenic control using CRISPR/Cas9. The 3 cell lines used to characterize the mutation were from an unaffected family member (“wild-type,” *NPPA*-WT) who did not carry the mutation and from the AF proband (“mutant,” *NPPA*-S64R) (Figure 1B). We generated the third cell line by genome correcting (GC) the *NPPA*-S64R mutation (*NPPA*-S64R-GC).

Our optimized EMM began immediately after glucose starvation (day 15) with iPSC-aCMs dissociated and replated onto fibronectin-coated tissue culture plates. After 3 days, we initiated EMM treatment. The addition of TID and FAs (palmitic and oleic acid) to our baseline culture media created a rich media that better mimics the physiological needs of mature aCMs. We enhanced the environment with ES, which began at a frequency of 2 Hz, with periodic increases of 1 Hz/d for 5 days (days 18–23) up to 6 Hz (19), with the increasing intensity designed to acclimate the iPSC-aCMs (20). The physiological level (2 Hz) of ES was restored on days 24–25, after which it was discontinued to prevent stress-induced cellular toxicity; however, TID and FA exposure remained (Figure 1A). Long-term assessment up to day 60 showed that the iPSC-aCMs reached optimal maturity at day 32 with plateauing of key ion channels and structural, FA metabolism, and mitochondrial oxidative phosphorylation genes (Supplemental Figure 1A). Separating the components of the EMM treatment into TID + FA only and ES only failed to achieve synergistic maturation in the EP, structural, and metabolic signature (Supplemental Figure 1B). Having established that iPSC-aCMs reached optimal maturity at day 32, we now refer to the matured cells as EMM iPSC-aCMs. Our immature iPSC-aCMs, which were also cultured to day 32 but did not undergo the EMM protocol, are referred to as “baseline” (Figure 1A).

### EMM enhances structural maturity.

We performed IF on baseline iPSC-aCMs, EMM iPSC-aCMs, and freshly isolated human aCMs (haCMs) from surgical samples to assess structural maturity (staining for α-actinin and cardiac troponin T, cTnT). Sarcomere organization improved with EMM treatment with uniform ultrastructural organization from the periphery to the perinuclear region (Figure 1C). EMM iPSC-aCMs were longer by 61.9% (baseline: 93.5 ± 19.8 μm vs. EMM: 151.4 ± 31.4 μm, *P* < 0.05), and the width was shorter by 45.6% (baseline: 58.1 ± 12.5 μm vs. EMM: 29.3 ± 9.1 μm, *P* < 0.05) (Figure 1D), with both not different from haCMs (length: 178.3 ± 37.5 μm; width: 48.7 ± 10.2 μm). Sarcomere length in EMM iPSC-aCMs increased by 23.4% (baseline: 1.75 ± 0.08 μm vs. EMM: 2.16 ± 0.03 μm, *P* < 0.05) and was comparable to the sarcomere length of haCMs (2.29 ± 0.04 μm; Figure 1E). Western blots showed increased expression of cTnT, cardiac troponin T I (cTnI), and sarco/endoplasmic reticulum calcium-ATPase (SERCA) in EMM iPSC-aCMs to a level not different from HAT (Figure 1, F and G). Assessing slow skeletal troponin I (ssTnI) expression showed that neither ssTnI nor cTnI was detected in the baseline iPSCs, but expression was markedly increased in EMM iPSC-aCMs. A potential metric of iPSC-CM maturity is the ratiometric isoform switch from ssTnI to cTnI, but reported studies focused on ventricular or heterogeneous populations of iPSC-CMs (32). Enhancements in structural maturity were replicated in the 2 additional cell lines (Supplemental Figure 2).

### EMM enhances metabolic maturity.

**Figure 2:**
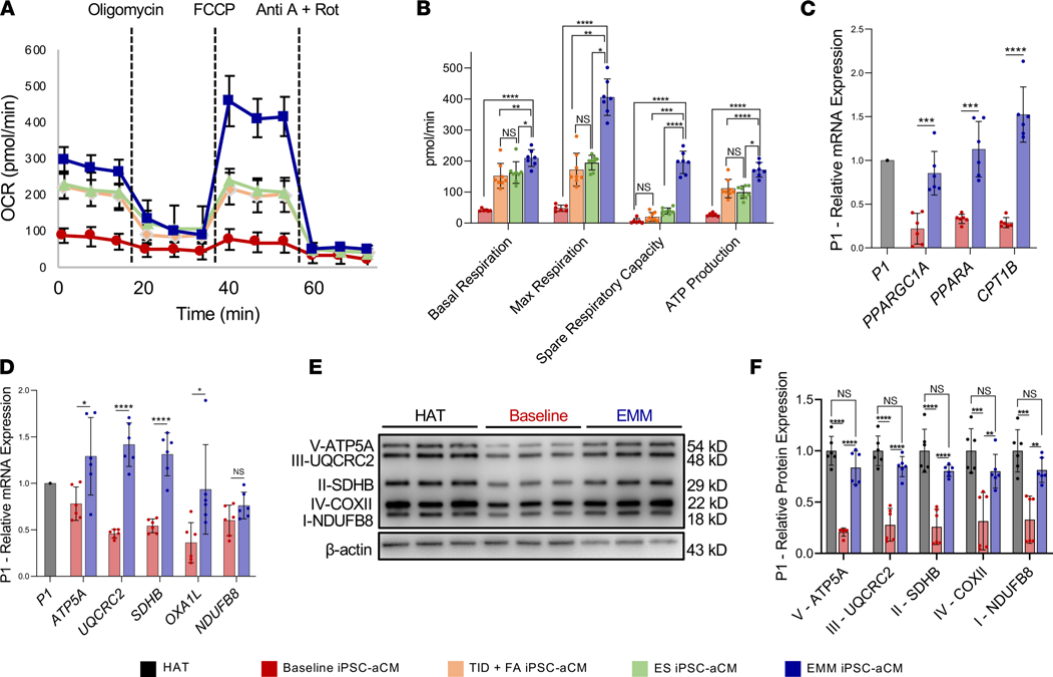
EMM enhances the metabolic maturity of iPSC-aCMs. (**A** and **B**) Real-time oxygen consumption rate (OCR) measurements of baseline, TID+FA only, ES only, and EMM iPSC-aCMs by Seahorse XFe96 showed that EMM iPSC-aCMs exhibited the highest respiration rate under basal conditions and after mitochondrial uncoupling (2-way ANOVA with Bonferroni’s correction). (**C** and **D**) Molecular assessment of genes involved in FA oxidation (**C**) and mitochondrial metabolism (**D**) shows that EMM significantly upregulated expression of relevant genes to a level comparable to that of HAT from the same patient; (*n* = 3 batches, *n* = 2 biological replicates per batch) (nonparametric Mann-Whitney *U* test). (**E** and **F**) Western blots of mitochondrial oxidative phosphorylation proteins show that EMM significantly upregulated expression of each of the 5 mitochondrial complexes involved in mitochondrial oxidative phosphorylation; (*n* = 6) (2-way ANOVA with Bonferroni’s correction); *P < 0.05, **P < 0.01, ***P < 0.001, ****P < 0.0001.

We functionally assessed the improvement in oxidative capacity of the EMM iPSC-aCMs using the Seahorse XF assay (Mito Stress Assay) to measure oxygen consumption rate (OCR) (Figure 2, A and B). Quantifying OCR parameters revealed that EMM iPSC-aCMs demonstrated higher basal metabolism, ATP production, maximal respiration, and spare respiratory capacity compared with baseline iPSC-aCMs, as well as TID + FA only and ES only iPSCs. In P1, EMM iPSC-aCMs had approximately 400% higher basal respiration, approximately 750% higher maximal respiration, and approximately 2850% higher spare respiratory capacity (basal: 41.70 ± 4.06 [baseline] vs. 209.75 ± 27.18 [EMM] pmol/min/cell number; maximal: 47.89 ± 10.02 [baseline] vs. 405.58 ± 58.88 [EMM] pmol/min/cell number; spare: 12.08 ± 9.07 [baseline] vs. 195.83 ± 35.88 [EMM] pmol/min/cell number). We also observed a significant improvement in OCR parameters in the 2 additional cell lines (Supplemental Figure 3, A and B).

We corroborated this functional assessment of metabolic maturity by assessing transcriptomic and proteomic expression levels of oxidative phosphorylation complexes and FA oxidation. EMM increased transcription of peroxisome proliferator–activated receptor (*PPARGC1A*, *PPARA*) and FA transport genes (carnitine palmitoyltransferase 1B, *CPT1B*) (Figure 2C). EMM iPSC-aCMs showed increased mRNA expression when compared with baseline iPSC-aCMs and no difference compared to HAT from the same patient in the 5 mitochondrial oxidative phosphorylation complexes: NDUFB8 (NADH ubiquinone dehydrogenase subunit B8; complex I), SDHB (succinate dehydrogenase subunit B; complex II), UQCRC2 (ubiquinol-cytochrome C reductase protein 2; complex III), COX II (cytochrome c oxidase subunit II; complex IV), and ATP5A (ATP synthase; complex V) (Figure 2D). We observed significantly increased protein expression of each of the complexes in EMM iPSC-aCMs when compared with baseline, but protein expression in EMM iPSC-aCMs was not significantly different from that in HAT (Figure 2, E and F; and Supplemental Figure 3, C and D).

### EMM improves EP.

**Figure 3:**
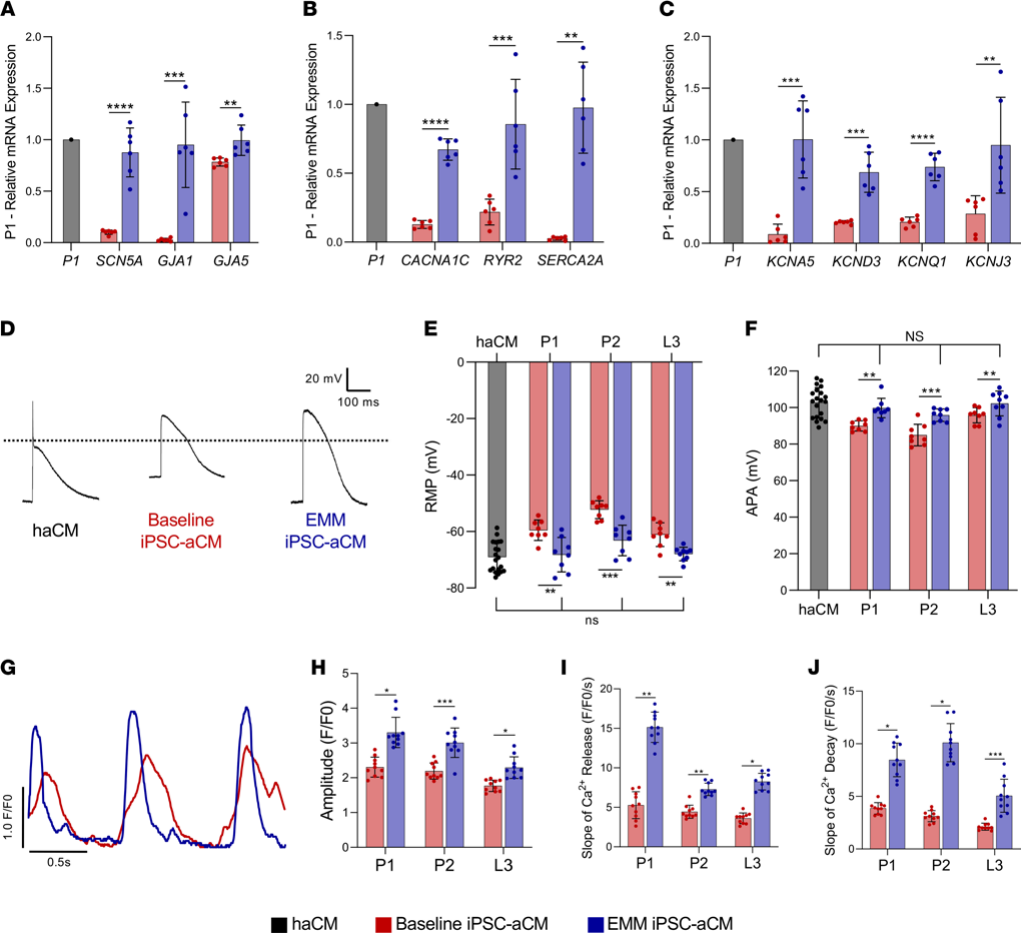
EMM enhances the EP, molecular, and calcium kinetic parameters of iPSC-aCMs compared with human adult aCMs from the same patient. (**A**–**C**) EMM increased expression of key ion channels involved in generation of the atrial AP of HAT, baseline iPSC-aCMs, and EMM iPSC-aCMs, focused on sodium channels and gap junctions (**A**), calcium handling genes (**B**), and potassium channels (**C**); (*n* = 3 batches, *n* = 2 biological replicates per batch) (nonparametric Mann-Whitney *U* test). (**D**–**F**) EMM optimized EP maturation: representative atrial APs in haCMs (left), baseline iPSC-aCMs (middle), and EMM iPSC-aCMs (right) (**D**); quantification of resting membrane potential (RMP) (**E**); and quantification of AP amplitude (APA) (**F**) (2-way ANOVA with Bonferroni’s correction). EMM iPSC-aCMs displayed an RMP significantly more hyperpolarized than baseline iPSC-aCMs, and EMM iPSC-aCM RMP was no longer different from the RMP of haCMs. APA was also significantly increased in EMM iPSC-aCMs compared with baseline iPSC-aCMs, and the APA of EMM iPSC-aCMs was also no longer different from the APA of haCMs. (**G**–**J**) EMM resulted in improved calcium kinetics: representative calcium kinetic tracings obtained using Fluo-4 (**G**) and quantification of amplitude (**H**), rate of Ca^2+^ release (**I**), and Ca^2+^ decay (**J**) comparing baseline iPSC-aCMs and EMM iPSC-aCMs. Calcium kinetics show that EMM iPSC-aCMs demonstrate higher calcium intracellular concentration, faster calcium release from SR, and faster calcium resequestration compared with baseline iPSC-aCMs. Each trace and quantification was average normalized (F/F0) from multiple peaks from each cell and each batch; (*n* = 2 batches, *n* = 5 cells per batch) (nonparametric Mann-Whitney *U* test); *P < 0.05, **P < 0.01, ***P < 0.001, ****P < 0.0001.

Adult aCMs, isolated from 20 donors undergoing cardiac surgery, underwent whole-cell current-clamping and had resting membrane potential (RMP), AP amplitude (APA), upstroke velocity, and APD measured. We compared the EP parameters in iPSC-aCMs with those from adult haCMs and showed increased transcriptomic expression of sodium, calcium, and potassium channels complemented by significant functional improvement across the 3 cell lines (Figure 3, A–C). RMP in haCMs was –69.62 ± 4.94 mV, with EMM iPSC-aCMs showing significantly hyperpolarized RMP as compared with baseline iPSC-aCMs (baseline: –52.3 ± 3.15 mV vs. EMM: –68.2 ± 6.10 mV; *P* < 0.01, *n* = 8) and no difference when compared to haCMs across the 3 cell lines (Figure 3, D and E). The APA in haCMs was 101.50 ± 9.13 mV, with the APA significantly increased in EMM iPSC-aCMs when compared with baseline (baseline: 85.0 ± 5.90 mV vs. EMM: 99.8 ± 5.32 mV; *P* < 0.01, *n* = 8) and no difference when compared to haCMs across the 3 cell lines (Figure 3, D–F). However, APD at 90% repolarization (APD_90_) and upstroke velocity were unchanged with EMM treatment across the 3 iPSC-aCM lines (Supplemental Figure 1, C and D).

We assessed the functional effect of EMM on iPSC-aCM calcium handling using fluorescent calcium dye Fluo-4-AM to measure calcium transients. EMM significantly increased the magnitude of calcium release from sarcoplasmic reticulum (SR) by 34.0% (baseline: 2.35 ± 0.27 fluorescence intensity/background fluorescence levels [F/F0] vs. EMM: 3.15 ± 0.16 F/F0, *P* < 0.01; Figure 3, G and H). EMM iPSC-aCMs displayed increased rate of Ca^2+^ release by 170.1% (baseline: 5.79 ± 2.04 F/F0/s vs. EMM: 15.64 ± 3.41 F/F0/s, *P* < 0.01; Figure 3, G–I). The rate of Ca^2+^ decay also increased by 124.7% (baseline: 3.80 ± 0.38 F/F0/s vs. EMM: 8.54 ± 2.09 F/F0/s, *P* < 0.05), with similar results across the 3 cell lines (Figure 3, G–J). We validated the functional improvement in calcium handling by measuring increases in transcription levels of key calcium handling genes (*CACNA1C*, *RYR2*, *SERCA2*; Figure 3B). Collectively, our data indicate that our EMM approach markedly enhanced iPSC-aCM calcium kinetics displaying a more adult-like signature.

### Enrichment of Gene Ontology pathways.

**Figure 4:**
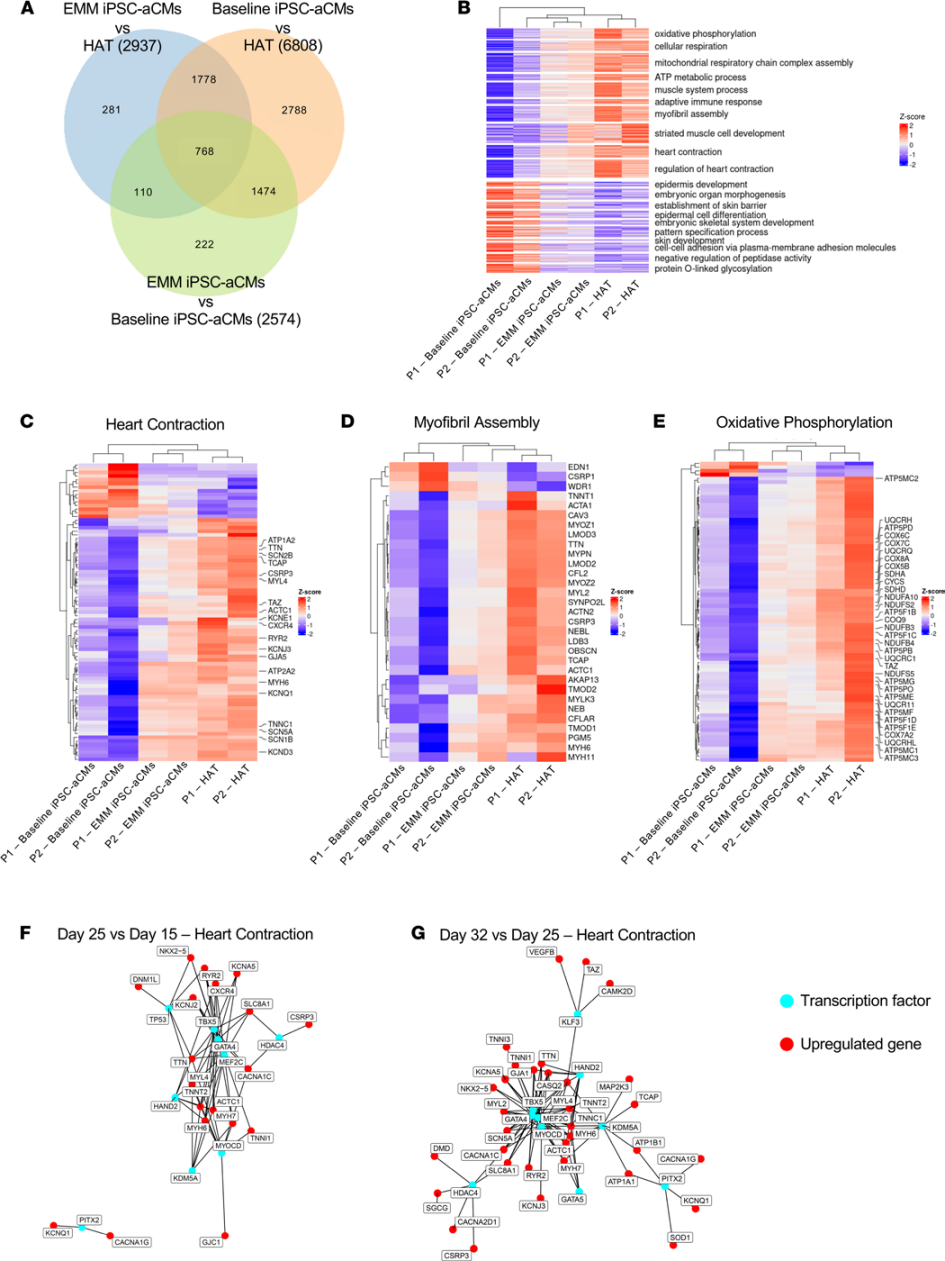
Unbiased transcriptomic analysis and gene regulatory network analysis identifies key Gene Ontology pathways and transcription factors mediating iPSC-aCM development and maturation. (**A**) Venn diagram showing the number of intersected upregulated differentially expressed genes (DEGs) among the 3 major comparisons: EMM iPSC-aCMs vs. HAT from the same patient, baseline iPSC-aCMs vs. HAT from the same patient, and EMM iPSC-aCMs vs. baseline iPSC-aCMs. (**B**) Heatmap of top upregulated and downregulated GO terms associated with DEGs comparing baseline iPSC-aCMs vs. EMM iPSC-aCMs vs. HAT from the same patients. The top GO terms upregulated were related to oxidative phosphorylation and mature methods of metabolism, myofibril assembly and structural development, and heart contraction. The top GO terms downregulated were related to embryonic and fetal processes. (**C**–**E**) Heatmaps of top upregulated and downregulated DEGs associated with the key GO pathways heart contraction (GO: 0060047) (**C**), myofibril assembly (GO: 0030239) (**D**), and oxidative phosphorylation (GO: 0006119) (**E**). (**F** and **G**) TF regulatory networks examining temporal development of TF regulation of the pathway heart contraction during maturation, comparing day 25 (midway through maturation) with day 15 (prior to initiation of maturation) (**F**) and day 32 (after maturation) versus day 25 (**G**). Increased target enrichment of upregulated genes overall and specific to each TF, as well as differential patterns of ion channel regulation at progressive time points, provide novel insights into temporal development and maturation of the pathway heart contraction.

We next performed an unbiased global transcriptomic analysis by RNA sequencing (RNA-Seq) of the P1 and P2 iPSC-aCM lines along with the corresponding HAT biopsied from the same patient. Comparing baseline iPSC-aCMs with EMM iPSC-aCMs showed that EMM treatment resulted in upregulation of 1257 differentially expressed genes (DEGs) and downregulation of 1317 (|LFC| ≥ 1, *q* < 0.05; LFC, log fold change) (Figure 4A and Supplemental Figure 4, A and B). Gene Ontology (GO) enrichment analysis mapped upregulated terms to muscle system process/contraction, heart process/contraction, mitochondrial metabolism/oxidative phosphorylation, and sarcomeric assembly/organization, with downregulated terms mapped to embryogenesis and fetal processes (Figure 4B). The downregulation signifies exit of iPSC-aCMs from the pluripotent and immature fetal state, with substantial progression toward an adult-like molecular signature. Comparing the EMM iPSC-aCMs with HAT from the same patient showed 926 genes upregulated genes and 2011 downregulated (|LFC| ≥ 1, *q* < 0.05), with GO enrichment analysis mapping to immunological processes (Supplemental Figure 5, A and B). Comparing the baseline iPSC-aCMs with HAT showed 3115 genes upregulated genes in HAT, and 3693 genes were downregulated (|LFC| ≥ 1, *q* < 0.05), with GO enrichment analysis mapping upregulated terms to mitochondrial metabolism, oxidative phosphorylation, muscle contraction, and myofibril assembly (Supplemental Figure 5, C and D).

We used an assessment algorithm to identify distinguishing biological pathways selected for the intersection of non-DEGs from the EMM iPSC-aCMs versus HAT (*q* > 0.05) with the DEGs of baseline cells versus HAT (|LFC| ≥ 1, *q* < 0.05). The enriched biological pathways included oxidative phosphorylation, mitochondrial respiratory chain complex assembly, ATP production, muscle/heart contraction, and myofibril assembly (Figure 4B) and showed that EMM iPSC-aCMs more closely resembled HAT. We repeated this assessment algorithm on 3 key GO pathways that reflect the targeted maturation parameters: heart contraction (GO: 0060047), myofibril assembly (GO: 0030239), and oxidative phosphorylation (GO: 0006119), which focus on EP, structural, and metabolic maturation, respectively (Figure 4, C–E, and Supplemental Figure 6). For heart contraction, DEGs that were upregulated in EMM iPSC-aCMs compared with patient-matched HAT included *KCNE1*, *KCNJ3*, *KCNQ1*, *MYL4*, *KCND3*, *GJA5*, *SCN5A*, *SCN1B*, *SCN2B*, *ATP1A2*, *RYR2*, *CASQ2*, *TNNC1*, and *TTN* (Figure 4C). Our analyses also revealed marked upregulation of potentially novel markers of maturation, such as tafazzin (*TAZ*), which catalyzes remodeling of immature cardiolipin to its mature form, and cardiac LIM protein (*CSRP3*), which has been implicated in mechano-signaling processes and actin dynamics (33). In myofibril assembly, DEGs that were upregulated in EMM iPSC-aCMs included *MYOM1*, *MYH6*, *TCAP*, *ACTC1*, and *TTN* (Figure 4D). For oxidative phosphorylation, the upregulated DEGs encompassed genes involved in the 5 ETC complexes: complexes I–V (Figure 4E).

### Time series analysis reveals transcription factor regulation of key biological pathways involved in aCM development.

We performed RNA-Seq of L3 EMM iPSC-aCMs as a time series to examine aCM development, selecting for 3 time points (Supplemental Figure 7). Day 15 of iPSC-aCM culture, immediately before EMM treatment, is the fetal point of CM development; day 25 is midway through maturation; and day 32 completes 2 full weeks of EMM treatment, representing the adult state. Comparing day 25 EMM iPSC-aCMs versus day 15 revealed differential upregulation of 576 genes and downregulation of 39 (|LFC| ≥ 1, *q* < 0.05). Comparing day 32 EMM iPSC-aCMs versus day 25 showed upregulation of 2477 genes and downregulation of 2533. We then determined the upstream TF regulators temporally related to maturation by performing Ingenuity Pathway Analysis (IPA) focusing on the same 3 key GO pathways (heart contraction, myofibril assembly, and oxidative phosphorylation), enabling us to identify potentially novel independent and integrated TF network regulation at each time point and within each GO pathway.

To examine the temporal maturation of the GO pathways, we selected transcription factors (TFs) that regulated the enriched DEGs important for maturation. For heart contraction, when comparing day 25 EMM iPSC-aCMs and day 15, we selected 7 TFs: *TP53,* *TBX5,* *GATA4,* *MEF2C,* *HAND2,* *KDM5A,* and *PITX2* (Figure 4F). When comparing day 32 EMM iPSC-aCMs and day 25, 10 TFs were selected: *TBX5*, *GATA4*, *MEF2C*, *MYOCD*, *HAND2*, *KDM5A*, *PITX2*, *HDAC4*, *KLF3*, *GATA5* (Figure 4G). We selected the TFs based on significant enrichment of the TF (*q* < 0.05) and then significant regulation of heart contraction, and further selection based on gene targets critical for AP generation and contraction. For structural maturity, examined using the GO pathway myofibril assembly with both time point comparisons (day 25 EMM iPSC-aCMs vs. day 15 and day 32 EMM iPSC-aCMs vs. day 25), we selected the same 7 TFs (*MEF2C*, *GATA4*, *HAND2*, *KDMA5*, *MYOCD,* *TBX5*, and *HDAC4*). The 7 TFs regulated enrichment of 10 genes at day 25 (Supplemental Figure 8A), but at day 32, the same 7 TFs expanded their enrichment target to 18 genes (Supplemental Figure 8B). We used the same selection algorithm as with heart contraction, selecting for significantly enriched TFs, then significant regulation of myofibril assembly, then further selection based on gene targets in sarcomere subunits, assembly, and interaction. To examine metabolic maturation using the oxidative phosphorylation GO pathway, after selecting significantly enriched TFs and those that significantly regulated oxidative phosphorylation, we selected TFs that targeted enriched DEGs that encoded each of the 5 mitochondrial complexes involved in oxidative phosphorylation. When comparing day 25 EMM iPSC-aCMs, and day 15 cells, we selected 5 TFs to examine: *PPARGC1A,* *PPARGC1B,* *KDM5A,* *HDAC5,* and *FOXO1* (Supplemental Figure 8C). When day 32 cells were compared to day 25 we selected 8 TFs to examine: *PPARGC1A*, *PPARGC1B*, *KDM5A*, *HDAC5*, *PITX2*, *FOXO1*, *ESRRA*, and *NRF1* (Supplemental Figure 8D).

### EMM maturation of AF-causing NPPA-S64R iPSC-aCMs revealed ion channel remodeling.

**Figure 5:**
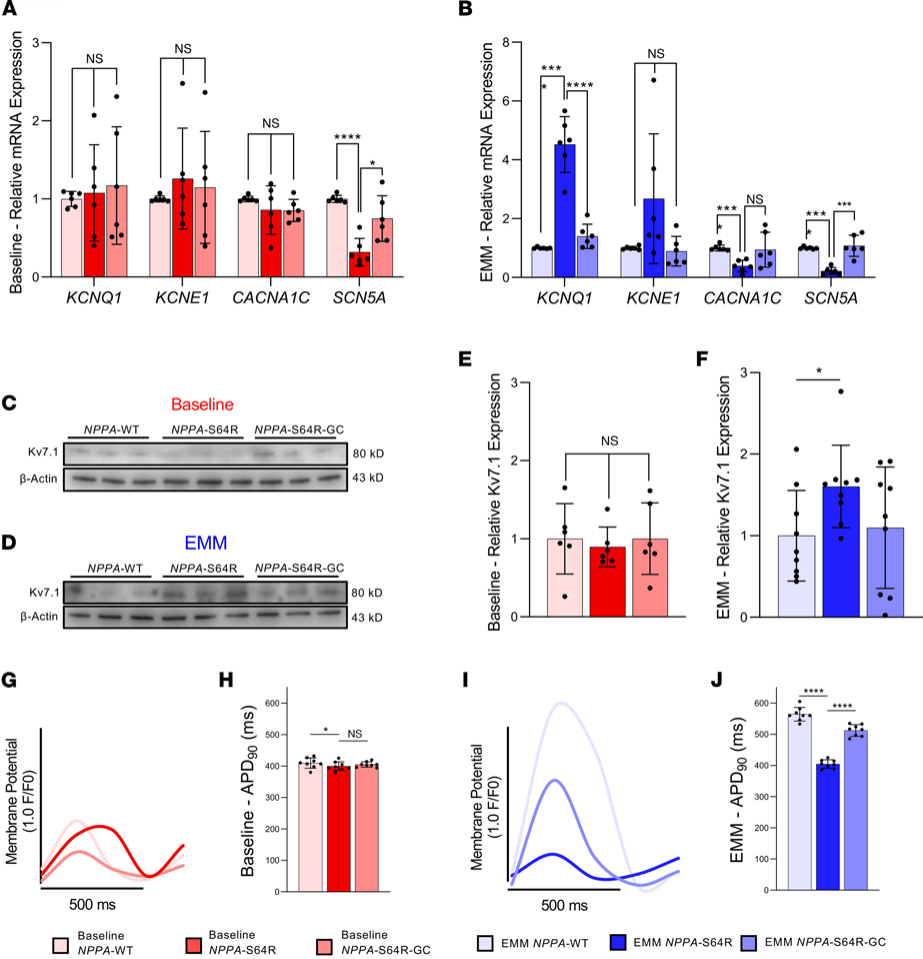
EMM iPSC-aCMs elucidate the underlying EP and molecular phenotype of the *NPPA*-S64R mutation. (**A** and **B**) Relative gene expression of *KCNQ1*, *KCNE1*, *CACNA1C*, and *SCN5A* for baseline *NPPA*-WT, *NPPA-*S64R, and *NPPA-*S64R-GC iPSC-aCMs (**A**) and relative gene expression of *KCNQ1*, *KCNE1*, *CACNA1C*, and *SCN5A* for EMM *NPPA*-WT, *NPPA-*S64R, and *NPPA-*S64R-GC iPSC-aCMs (**B**) (*n* = 2 batches, *n* = 3 biological replicates per batch) (2-way ANOVA with Bonferroni’s correction). (**C**–**F**) EMM enhances expression of cardiac potassium channel. Representative western blots for Kv7.1 for baseline *NPPA*-WT, *NPPA-*S64R, and *NPPA-*S64R-GC iPSC-aCMs (**C**) and EMM *NPPA*-WT, *NPPA-*S64R, and *NPPA-*S64R-GC iPSC-aCMs (**D**), and quantification of Kv7.1 expression for baseline *NPPA*-WT, *NPPA-*S64R, and *NPPA-*S64R-GC iPSC-aCMs (red) and EMM *NPPA*-WT, *NPPA-*S64R, and *NPPA-*S64R-GC iPSC-aCMs (blue) (**E** and **F**). Western blot analysis shows that Kv7.1 expression is increased in EMM *NPPA-*S64R compared with EMM *NPPA*-WT. However, Kv7.1 expression was not increased in baseline *NPPA*-S64R compared to baseline *NPPA*-WT; (*n* = 3 batches, *n* = 3 biological replicates per batch) (1-way ANOVA with Bonferroni’s correction). (**G**–**J**) EMM shortens the APD in *NPPA-S64R*: representative optical AP recordings of baseline *NPPA-*WT, *NPPA-*S64R, and *NPPA*-S64R-GC iPSC-aCMs with voltage-sensitive dye VF2.1Cl (**G**) and APD_90_ quantification (**H**) and representative optical AP recordings of EMM *NPPA*-WT, *NPPA*-S64R, *NPPA-*S64R-GC iPSC-aCMs with voltage-sensitive dye VF2.1Cl (**I**) and APD_90_ quantification (**J**). EMM *NPPA*-S64R iPSC-aCMs display marked shortening of the APD compared with EMM *NPPA*-WT. Baseline *NPPA*-S64R iPSC-aCMs do not demonstrate such a striking shortening of the APD compared to EMM *NPPA*-WT (1-way ANOVA with Bonferroni’s correction); *P < 0.05, **P < 0.01, ***P < 0.001, ****P < 0.0001.

We applied our EMM approach to characterize the first non-ion channel gene that has been linked with familial AF (10). We generated iPSC-aCMs from a family carrying an *NPPA-*S64R mutation and an isogenic control using CRISPR/Cas9 (Figure 1B and Supplemental Figure 9). Comparing *NPPA-*WT, *NPPA-*S64R, and *NPPA-*S64R-GC, iPSC-aCMs displayed no difference in the mRNA expression of *KCNQ1* and *KCNE1* (Figure 5A). There was also no difference in the expression of Kv7.1 or its β subunit (Figure 5, C and E). However, applying EMM to each of these cell lines showed significant upregulation in *KCNQ1* and *KCNE1* expression (Figure 5B), as well as significantly increased expression of Kv7.1 (Figure 5, D and F) in EMM *NPPA*-S64R iPSC-aCMs.

We then used optical voltage mapping to assess functional EP parameters (Figure 5, G–I) and showed that baseline *NPPA-*S64R iPSC-aCMs exhibited no significant difference in APD_90_ (414.92 ± 20.51 ms [baseline, *NPPA-*WT] vs. 397.28 ± 20.70 ms [baseline, *NPPA-*S64R] vs. 405.00 ± 24.51 ms [baseline, GC]; Figure 5, G and H). APD_90_ shortening was more pronounced in the mature *NPPA-*S64R iPSC-aCMs versus the mature *NPPA-*WT iPSC-aCMs, and this shortening was abrogated in the mature *NPPA-*S64R-GC iPSC-aCMs (561.80 ± 23.11 ms [EMM *NPPA-*WT] vs. 402.05 ± 32.05 ms [EMM *NPPA-*S64R] vs. 509.06 ± 45.35 ms [EMM *NPPA-*S64R-GC]; Figure 5, I and J).

**Figure 6:**
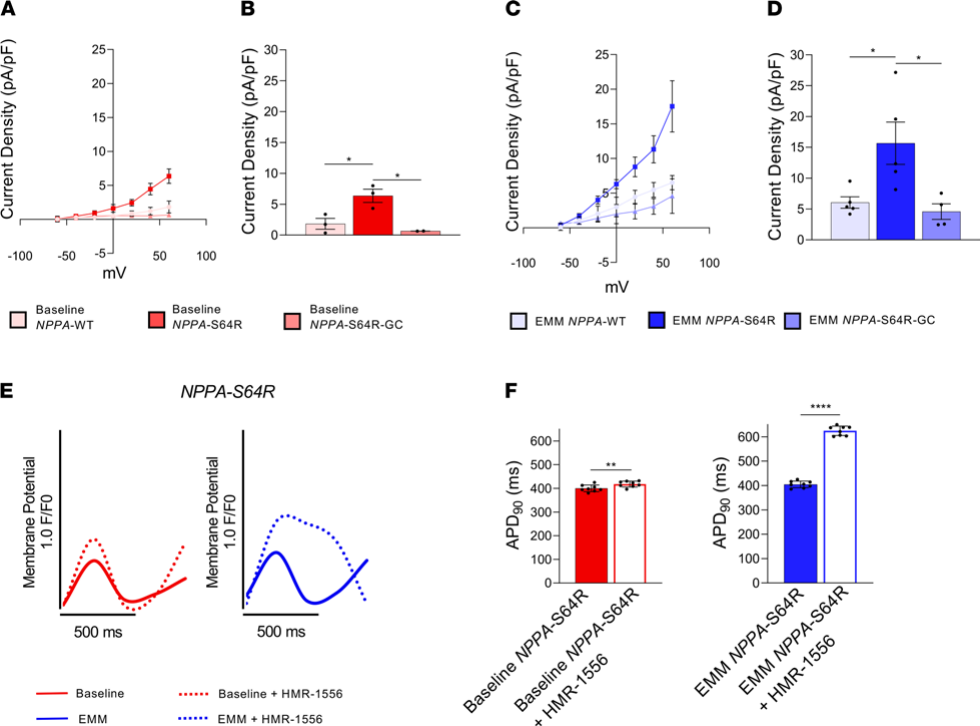
EMM iPSC-aCMs reveal EP mechanism of *NPPA*-S64R mutation. (**A**–**D**) I_Ks_ density from whole-cell voltage-clamp revealed increased I_Ks_ in EMM *NPPA-*S64R iPSC-aCMs. (**A** and **B**) I_Ks_ current-voltage (I-V) curves (mean ± SEM) (**A**) and quantification of I_Ks_ densities at +60 mV (**B**) for baseline *NPPA*-WT, *NPPA-*S64R, *NPPA*-S64R-GC iPSC-aCMs, examining *n* = 2–3 cells per group (1-way ANOVA with Bonferroni’s correction). (**C** and **D**) I_Ks_ current-voltage (I-V) curves (mean ± SEM) (**C**) and quantification of I_Ks_ densities at +60 mV (**D**) for EMM *NPPA*-WT, *NPPA-*S64R, and *NPPA*-S64R-GC iPSC-aCMs, examining *n* = 4–5 cells per group (1-way ANOVA with Bonferroni’s correction). (**E**) Representative optical AP recordings with voltage-sensitive dye VF2.1Cl on *NPPA*-S64R iPSC-aCMs, comparing baseline iPSC-aCMs (red) with EMM iPSC-aCMs (blue). Dotted recordings depict AP changes with infusion of 1 μM HMR-1556 (selective I_Ks_ blocker). (**F**) APD_90_ quantification of *NPPA*-S64R iPSC-aCMs, comparing baseline iPSC-aCMs (red) with EMM iPSC-aCMs (blue). APD_90_ prolongation with administration of I_Ks_ blocker in EMM *NPPA*-S64R iPSC-aCMs is much more striking when compared with that of baseline *NPPA*-S64R iPSC-aCMs; *n* = 2 batches, *n* = 4 cells per batch; *P < 0.05, **P < 0.01, ***P < 0.001, ****P < 0.0001 (nonparametric Mann-Whitney *U* test).

To determine the cellular mechanism by which the *NPPA* mutation caused AF, we measured I_Ks_ by whole-cell voltage-clamping_._ The increase in I_Ks_ density was significantly more pronounced in EMM *NPPA-*S64R iPSC-aCMs vs. EMM *NPPA*-WT iPSCs (Figure 6, C and D) when compared with baseline *NPPA*-S64R and baseline *NPPA*-WT iPSC-aCMs (Figure 6, A and B, and Supplemental Figure 10). I_Ks_ density in EMM *NPPA*-S64R-GC iPSC-aCMs was comparable to EMM *NPPA-*WT. When we applied the selective I_Ks_ blocker HMR-1556 to the immature *NPPA-*S64R iPSC-aCMs, APD_90_ was only marginally increased (397.28 ± 20.70 ms [baseline] vs. 420.12 ± 24.75 ms [EMM]; Figure 6, E and F). In contrast, applying it to mature *NPPA-*S64R iPSC-aCMs markedly prolonged the APD_90_ (402.05 ± 32.05 ms [baseline] vs. 626.76 ± 64.32 ms [EMM]; Figure 6, E and F; and Supplemental Figure 11, A–D).

### EMM maturation of NPPA-S64R iPSC-aCMs is associated with mitochondrial defects.

**Figure 7:**
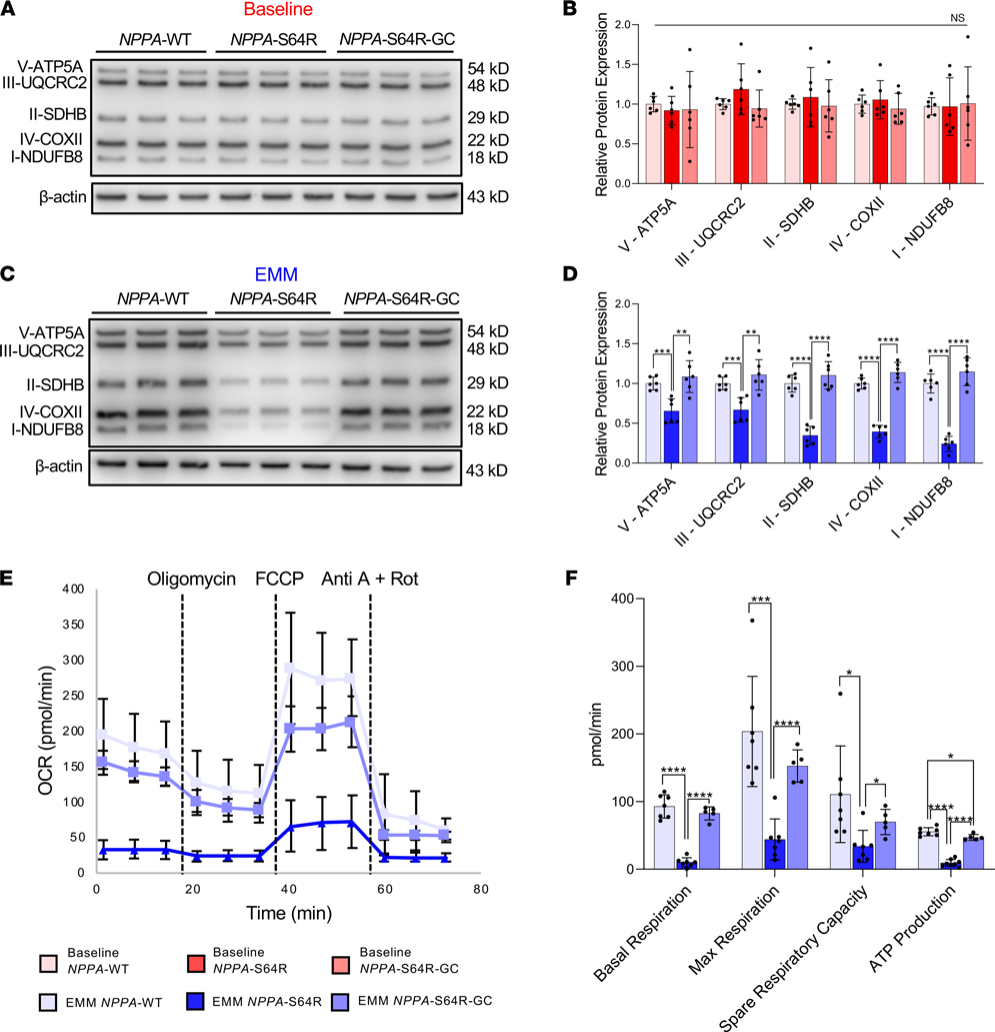
Metabolic substrate underlying *NPPA-*S64R mutation. (**A** and **B**) Western blot of mitochondrial oxidative phosphorylation proteins in baseline *NPPA*-WT, baseline *NPPA*-S64R, and baseline *NPPA*-S64R-GC iPSC-aCMs shows no significant difference in the expression of any of the 5 complexes involved in the electron transport chain (ETC). (**C** and **D**) Western blot of mitochondrial oxidative phosphorylation proteins in EMM *NPPA*-WT, EMM *NPPA*-S64R, and EMM *NPPA*-S64R-GC iPSC-aCMs shows that expression of the each of the 5 complexes is significantly reduced in *NPPA*-S64R iPSC-aCMs, indicating the necessity of utilizing matured iPSC-aCMs; (*n* = 2 batches, *n* = 3 biological replicates per batch). (**E** and **F**) Real-time oxygen consumption rate (OCR) measurements of EMM *NPPA*-WT, EMM *NPPA*-S64R, and EMM *NPPA*-S64R-GC iPSC-aCMs show that *NPPA*-S64R iPSC-aCMs display significantly reduced respiration rate under basal conditions and after mitochondrial coupling. (*n* = 2 batches, *n* = 2–4 biological replicates per batch); *P < 0.05, **P < 0.01, ***P < 0.001, ****P < 0.0001 (2-way ANOVA with Bonferroni’s correction).

Since AF has been associated with altered cardiac energetics in humans (34, 35), we hypothesized that atrial metabolism was reduced in *NPPA*-S64R iPSC-aCMs. We used Western blotting to assess proteomic expression of the 5 mitochondrial complexes involved in oxidative phosphorylation. In baseline iPSC-aCMs, there was no change in the expression level of any complex (Figure 7, A and B). However, infusing the mutant ANP produced by the *NPPA*-S64R mutation into P1 EMM iPSC-aCMs revealed selective downregulation in the expression of complex I and complex IV (Supplemental Figure 11, E and F), suggesting that mature iPSC-aCMs were necessary to unmask the metabolic defects. When we examined the WT, mutant, and GC cell lines, EMM *NPPA*-S64R iPSC-aCMs displayed an overall reduction in all 5 complexes (Figure 7, C and D), indicating that a defect in complex I is responsible for the early development of the metabolic substrate for AF, with this defect leading to a reduction in the expression of the ETC. To assess overall cellular bioenergetics, we functionally assessed OCR and demonstrated that EMM *NPPA*-S64R iPSC-aCMs displayed significantly reduced oxidative capacity, and EMM *NPPA*-S64R-GC displayed a near-complete recovery in oxidative capacity to that in WT (Figure 7, E and F). These functional data correlate with the function of complex I and the selective downregulation of complex I and complex IV in the *NPPA*-S64R mutation.

## Discussion

Cardiac tissue is the best model to identify the cellular mechanisms of AF but is rarely available from patients due to the risks and complexity of the invasive procedure. Limitations of existing in vitro and in vivo models of AF necessitate the development of alternate approaches, which generate mature aCMs suited for the assessment of AF pathophysiology. The emergence of human iPSC-CMs serves as a potentially faithful model of AF, but their physiological and functional immaturity limits their fidelity at identifying proximate mechanisms. Currently, there are limited data on the maturation of iPSC-aCMs because of poor characterization; limited understanding of the intersection of EP, structural, and metabolic parameters; and an inability to define the optimal level of maturation necessary to model AF. The lack of maturity of human iPSC-aCMs is one potential explanation for the knowledge gap in understanding the cellular mechanisms by which *NPPA* mutations cause AF.

Our potentially novel EMM approach utilizes natural stimuli during gestation and postnatal development, synergistically replicating the natural and integrated EP, structural, and metabolic development in order to enhance iPSC-aCM maturity. While biochemical stimulation with hormones (TID), FA supplementation, and ES has been applied individually to mature iPSC-CMs, this is the first study to our knowledge that combined all 3 approaches with the goal of comprehensively maturing iPSC-aCMs. Importantly, most maturation protocols either have focused on a ventricle-specific population or have utilized a heterogeneous CM population. To model a mutation associated with AF, it is critical to utilize a cellular model composed of primary aCMs. As there is early divergence in atrial and ventricular development during gestation, we postulated that the aCMs would respond differently to the maturation approaches compared with existing data on ventricular CMs, and that EMM was both additive and synergistic in enhancing iPSC-aCM maturity. In 2 patient models, we used individual HAT obtained from the same patient from whom the iPSC-aCMs were derived to define the benchmark for maturation. Thus, we were able to show that the high level of maturity achieved with our EMM approach can be applied to multiple patient-derived cells. Unbiased transcriptomic analysis performed in tandem with a robust time series uncovered key transcription factors regulating the EP, structural, and metabolic development of the atria from the early fetal to adult stage, enabling us to identify independent and integrated regulatory networks for important GO pathways at distinct stages of atrial maturation. Finally, modeling the first non-ion channel gene (*NPPA*) identified as a cause of AF using mature iPSC-aCMs showed enhanced I_Ks_ and metabolic defects when compared with immature iPSCs and established a mechanistic link with the genetic substrate for AF in vitro.

This is the first study to our knowledge to examine the combinatorial effect of biochemical stimulation, FA supplementation, and ES. To optimize our approach for an atrium-specific cellular population, we first cultured the iPSC-aCMs to day 60 to examine the targeted transcriptomic profile of ion channel, calcium handling, structural, and metabolic genes. We discovered that the EMM iPSC-aCMs failed to mature further past day 32. Second, we determined the optimal time to initiate maturation, with a previous study reporting a small window of cellular plasticity during which iPSC-CMs would respond to maturation strategies (19). We determined that the optimal time to initiate our EMM protocol was immediately after replating the iPSC-aCMs onto fibronectin (day 18). Third, we determined the duration of time needed for ES. Although ventricular iPSC-CMs handle ES for several weeks (19), iPSC-aCMs in our ES setup only tolerate the increasing intensity regimen for 1 week beyond which they develop cytotoxicity. Thus, we increased the frequency of ES over 5 days, returned to a physiological frequency for 2 days, and stopped beyond that while continuing TID and FA. Fourth, to test the hypothesis that combining TID, FA supplementation, and ES enhanced the maturity of iPSC-aCMs, we examined the effects of TID + FA only and ES only. We showed that individually TID + FA and ES minimally enhanced atrial maturity when compared to immature iPSC-aCMs and patient-specific aCMs. Both TID + FA and ES alone only partially increased the expression of key membrane-regulating genes (*CACNA1C*, *KCNA5*, *KCNQ1*, *RyR2*, and *SCN5A*). In contrast, combining all 3 physiological cues resulted in no differences in gene expression when compared to adult atrial tissue from the same patient. Furthermore, our EMM protocol enhanced structural maturity (*TNNT2*) and metabolic maturity (*CPT1B* and *PPARA*). Biochemical stimulation, bioenergetic conditioning, and ES acting simultaneously are key physiological cues that replicate atrial myocardial development. We postulate that our EMM approach is both additive and synergistic for enhancing iPSC-aCM maturity.

The mechanism by which the *NPPA*-S64R mutation enhanced the expression and function (I_Ks_) of the potassium channel and caused metabolic defects remains unclear. One postulated mechanism is modulation of upstream regulators such as TFs by *NPPA*. Our transcriptomic network analysis showed that PITX2 is the only common TF that links *NPPA*, *KCNQ1*, and *NDUFB8*. Pitx2 directly regulates genes encoding ion channels, and reduced expression is associated with increased susceptibility to AF (36, 37). Another possible mechanism is that the *NPPA*-S64R mutation itself creates an EP substrate for AF. We show that infusion of the mutant peptide produced by the *NPPA*-S64R mutation modulates the expression of ETC proteins, especially complex I. It is likely that the mutant peptide not only causes mitochondrial defects but also regulates ion channel expression, especially Kv7.1. We hypothesize that altered expression of *KCNQ1*/Kv7.1 in conjunction with metabolic defects create an EP substrate for AF. However, our data are hypothesis generating and additional experiments are warranted.

Modeling rare mutations in familial AF kindred using immature iPSC-aCMs has provided some insights into the underlying pathophysiology of AF and identified patient-specific mechanisms (16, 38). However, with the advent of advanced engineering techniques to further mature iPSC-aCMs, we predict that all future studies modeling genetic variants associated with AF will use mature iPSC-aCMs (39). Currently, pharmacological response to antiarrhythmic therapy is highly variable because of incomplete understanding of the pathophysiological mechanisms and our inability to predict responses in individual patients (40). Even small differences in baseline EP properties such as the RMP may modulate response to antiarrhythmic drugs, with Syeda et al. (41) reporting that flecainide was more efficacious in suppressing AF in mice with reduced Pitx2c expression when the RMP was more depolarized. We showed that EMM iPSC-aCMs significantly hyperpolarized the RMP as compared with baseline iPSC-aCMs, and there was no difference when compared to haCMs (Figure 3, D and E).

Our EMM approach improved many AP properties, but the APD and upstroke velocity remained unchanged. One potential explanation is that EMM did not fully mature all potassium channels, especially the transient outward potassium current (I_to_). Representative atrial APs from the baseline and EMM iPSC-aCMs appear to display reduced function of I_to_, which is a strong modulator of the atrial APD (42). While EMM treatment increased mRNA expression of *KCND3*, the gene encoding I_to_, the gating mechanisms and activation/inactivation kinetics of the channel may not yet be functionally mature. Despite optimal improvement in transcriptomic maturity, we postulate that culturing iPSC-aCMs beyond day 32 may be necessary to not only enhance functional maturity of sodium and transient outward potassium channels but also generate atrial APs that more closely resemble those from human atria. A second reason for why the APD and upstroke velocity may have remained unchanged with EMM is altered activation and inactivation kinetics of I_to_. Impaired inactivation gating of the channel has been shown to decrease cardiac APD (43). EMM increased the expression of the sodium channel, but failure to increase the upstroke velocity suggests reduced sodium current (I_Na_) or possibly altered gating. Prolonged culture may also enhance I_Na_. While the APD and upstroke velocity remained unchanged, our EMM approach successfully increased I_Ks_, enabling us to uncover the underlying genetic and molecular mechanisms by which the *NPPA*-S64R mutation causes AF.

Our study provides important insights into cardiac ion channel, sarcomeric, and metabolic development. Examining the temporal and interrelated relationships of TF networks in atrial maturation may provide valuable insights into phenotype-genotype relationships and disease progression. EP phenotypes need to be integrated with sarcomeric maturation because they form the basis of electromechanical contraction of the atria. Our analyses suggest that EMM enhances activation of key TFs such as *MEF2C*, *GATA4*, *HAND2*, and *HDAC4*, which are known to modulate sarcomere formation (44). The increased enrichment of sarcomeric genes was accompanied by upregulation of DEGs and the number of isoforms for each genetic family encoding the mitochondrial ETC during EMM, including multiple isoforms of complexes I–V. Our data suggest that the increased reliance on mitochondrial oxidative phosphorylation as the primary source of ATP production may be due to increased target enrichment of select TFs, including *PPARGC*, *PITX2*, *FOXO1*, and *HDAC5* (45). This results in increased activity and efficacy of each ETC complex, which collectively improves bioenergetic maturation that is necessary for sustained and efficient atrial contraction.

Using mature iPSC-aCMs, we modeled an *NPPA*-S64R mutation associated with AF (29). Functional characterization of mutant *NPPA*-S64R augmented I_Ks_, an effect predicted to shorten the atrial APD. Modeling studies also supported augmented I_Ks_ with AP shortening altering the L-type calcium current, a common AF mechanism. Immature iPSC-aCMs did not exhibit differences in ion channel expression when compared to *NPPA-*WT or the *NPPA-*S64R cell lines and therefore demonstrated only marginal reduction in the APD, thus making it impossible to uncover the AF mechanism or develop therapies targeting the EP substrate in patients carrying this mutation. In contrast, mature iPSC-aCMs exhibited not only marked increase in *KCNQ1* expression and function (I_Ks_) but also significantly shortened APD in *NPPA*-S64R iPSC-aCMs. Importantly, an I_Ks_-specific blocker (HMR-1556) had minimal effect in prolonging the APD in *NPPA-*WT and *NPPA-*S64R immature iPSC-aCMs, whereas applying it to the EMM *NPPA-*WT and *NPPA*-S64R iPSCs markedly prolonged the APD. Our results demonstrated that therapeutic targeting of I_Ks_ could be a novel therapeutic approach in patients with AF carrying the *NPPA*-S64R mutation.

Increasing evidence suggests that mitochondria, by providing ATP via the oxidation of FAs and other substrates, play a critical role in maintaining normal electrical and mechanical function of the heart (46, 47). A supply-demand imbalance in the production of high-energy phosphate compounds and metabolic defects can affect electrical activity through ion channel remodeling, oxidative stress, and modulation of cell death, thereby creating a substrate for arrhythmias (48, 49). However, studying oxidative phosphorylation in human AF is challenging because atrial tissue is rarely available, and confounding conditions associated with AF, such as hypertension, aging, and heart failure, can themselves lead to mitochondrial dysfunction. It also remains unclear if defects in mitochondrial energetics are causative or a consequence of AF (50). While oxidative stress plays an important role in AF pathogenesis, few studies have determined if oxidative stress causes mitochondrial dysfunction with impairment in the ETC activity in human atria (35). We examined the metabolic dysfunction associated with the *NPPA*-S64R mutation. Immature *NPPA*-WT, *NPPA*-S64R, and *NPPA-*S64R-GC iPSC-aCMs failed to exhibit differences in expression of the ETC complexes. However, externally infusing the mutant ANP produced by the *NPPA*-S64R mutation onto control (nondiseased) EMM iPSC-aCMs showed that complexes I and IV were selectively downregulated. This suggested that utilizing matured iPSC-aCMs is necessary to unmask the metabolic defects. The selective downregulation of complex I is particularly interesting since this complex is responsible for assembling the other respirasomes in the mitochondria. Thus, a reduction in complex I may lead to overall disassembly and intercomplex instability. When we examined EMM *NPPA*-WT, *NPPA*-S64R, and *NPPA*-S64R-GC iPSC-aCMs, we showed for the first time to our knowledge that the ETC functional activity and overall cellular oxidative capacity were reduced in *NPPA*-S64R iPSC-aCMs with downregulation of all complexes. This indicates that dysfunction of complex I is responsible for the early development of the metabolic substrate for AF, with this defect leading to a reduction in the overall expression of the ETC complexes. While the role of *NPPA* in the maintenance of mitochondrial function and expression of mitochondrial genes remains unknown, collectively, our data in EMM iPSC-aCMs support our hypothesis that ion channel remodeling in conjunction with metabolic defects created an EP substrate for AF. By unveiling the critical role of ANP-induced mitochondrial dysfunction in mature iPSC-aCMs, our work paves the way for the future investigation of personalized therapies targeting CM metabolism or ANP signaling in patients carrying the *NPPA* mutation. Such future therapies that restore aCM mitochondrial function could be used in high-risk individuals either to prevent the development of AF or be added as adjunctive therapy for AF rhythm control.

Our EMM approach, focused on integrated EP, structural, and metabolic maturation of iPSC-aCMs, unmasked the mechanistic link to the EP and metabolic substrate of AF induced by the *NPPA*-S64R mutation. While other approaches such as heart-on-a-chip and bioprinting (51) are currently under development, the monolayer nature of our model is especially advantageous for studying cellular EP and high-throughput drug screening using a multiwell plate-based format that is the workhorse in the pharmaceutical industry. Since AF is also a multicellular disease, the mature iPSC-aCM model we developed can serve as the foundation to investigate cellular crosstalk in disease progression by coculturing with nonparenchymal cells, the influence of environmental stimuli such as shear flow, and changes to overall atrial conduction (52).

In summary, using an EMM approach of physiologically inspired cues, we synergistically enhanced the EP, structural, metabolic, and molecular maturity of human iPSC-aCMs. For the first time to our knowledge, we have utilized a comprehensive maturation approach to successfully model the first non-ion channel gene identified as a cause of AF, establishing a mechanistic link with the genetic substrate and elucidating the underlying EP and molecular mechanisms of this common and morbid arrhythmia.

## Methods

### Isolating aCMs from HAT.

We obtained adult HAT biopsies during cardiothoracic surgery through the UIC AF Registry. HAT was transported in warmed EDTA, and aCMs were isolated using the Langendorff-free protocol as described (53). HAT was injected sequentially with EDTA, perfusion buffer, and collagenase buffer, 3 times each, with a flow rate of 2 mL/min using 30 G hypodermic needles. The tissue was gently sheared to dissociate aCMs from the tissue, followed with stop buffer in a 50 mL beaker to inhibit enzymatic activity. A warmed magnetic stirrer was added to the beaker and stirred for 15 minutes at low speed. The stirred cellular suspension was passed through a 200 μm mesh filter, and the supernatant was collected. The supernatant was centrifuged at 450*g* for 9 minutes, and the cell pellet was resuspended in 4 mL of culture media.

### Reprogramming PBMCs into iPSCs.

PBMCs were isolated using tubes containing EDTA and Ficoll and cultured using the Stanford Biobank protocol as previously described (16, 30). PBMCs were cultured in STEMPro034 SFM (Gibco) medium with cytokines at a density of 2 × 10^5^ cells/mL, for 7 days. The cells were transduced by Sendai virus using the CytoTune-iPS 2.0 Sendai Virus Reprogramming Kit (Thermo Fisher Scientific), delivered at an MOI of 5-5-3 (KOS MOI = 5, hc-Myc MOI = 5, hKlf4 MOI = 3) without polybrene, to a final total volume between 0.4 and 1 mL. Cells and viruses were centrifuged at 300*g* for 15 minutes at room temperature. Each PBMC pellet was resuspended in complete SFEM II (STEMCELL Technologies) with cytokines and transferred to a well of a 24-well plate. After 3–4 weeks, the colonies were visualized and manually picked. The iPSC colonies were maintained on Matrigel-coated wells in mTeSR1. For karyotyping, iPSCs at passage 4 were analyzed by G-band karyotyping at WiCell Research Institute.

### NPPA gene editing.

iPSCs heterozygous for NPPA p.S64R were genome corrected using CRISPR/Cas9. Briefly, allele-specific guide RNAs (gRNAs; 5′-tgagccgaatgaagaagcgg-3′) were designed with the Crispor program (http://crispor.tefor.net) to target exon 2 containing the mutant allele. iPSCs were treated with 10 μM Y-27632 for 24 hours pre-electroporation. For GC, iPSCs were dissociated into single cells, then electroporated with an ribonucleoprotein complex of Cas9 and single gRNA along with single-stranded oligodeoxynucleotides containing the WT template using the Neon Transfection System (Invitrogen). After electroporation, cells were transferred into 1 well of a Matrigel-coated 24-well plate containing 500 μL of mTesR (STEMCELL Technologies) with 10 μM Y-27632 (Selleckchem). After several days of expansion, half the cells were used to analyze editing efficiency with next-generation sequencing (NGS) amplicon sequencing. Upon confirming editing efficiency, 96 individual cells were manually sorted to establish single-cell colonies. Afterward, 25 edited clones, as well as 2 unedited clones exposed to the genome-editing pipeline but remaining unmodified, were selected for expansion, and NGS was used to verify gene correction and absence of off-target edits.

### Human iPSC culture and iPSC-aCM differentiation.

iPSCs were seeded at an initial density of 500,000 cells/well on 6-well plates coated with human recombinant vitronectin (Gibco) and then cultured in mTesR media with daily media exchanges until 80%–90% confluent. After washing the iPSCs with Dulbecco’s PBS (DPBS; Gibco) without Ca^2+^ or Mg^2+^, differentiation was initiated using the Cardiomyocyte Differentiation Kit (Gibco) following manufacturer-recommended procedures. To guide iPSC-CMs into an atrial subtype, on day 5 (D5), the cells were treated with 1 μM/all-trans retinoic acid (MilliporeSigma) for 4 days with media changes every 2 days (54). The cellular population was purified using glucose starvation and lactate replacement with contracting monolayers visualized between D9 and D12.

Between D14 and D16, the iPSC-aCMs were dissociated into single cells and replated onto fibronectin-coated (FBN-coated) plates by incubating in DPBS without Ca^2+^ or Mg^2+^ for 20 minutes in 37°C, followed by 5 minutes in TrypLE Express (Gibco) in 37°C, followed by 20 minutes in 25 μg/mL of Liberase (Roche) in 37°C. After gentile aspiration, the cells were centrifuged for 5 minutes at 500*g*, then resuspended in Cardiomyocyte Maintenance Media (Gibco) supplemented with 10% FBS. D18–D32, the base media was supplemented with 100 nM T3 (MilliporeSigma), 100 ng/mL insulin-like growth factor-1 (PeproTech), 1 μM dexamethasone (Biogems), and palmitic acid (50 μM) and oleic acid (100 μM) bound to BSA.

### IF and analysis.

Isolated haCMs were stained using α-actinin and cTnT using a nonadherent IF protocol performing all steps in suspension in a microcentrifuge tube, and replacing all centrifugation steps with a waiting period to allow cells to settle naturally into a pellet. For cultured iPSC-aCMs using an adherent protocol, the cells were first dissociated and replated at a low density onto confocal grade polymer-bottom dishes (ibidi), then recovered for 2 days. For both isolated haCMs and cultured iPSC-aCMs, the cells were washed with PBS, fixed with 4% paraformaldehyde in 37°C for 10 minutes, permeabilized using 0.1% Triton X-100 for 15 minutes, and blocked with 3% BSA for 1 hour. Primary antibody was diluted at a 1:200 in 0.1% BSA and incubated in 0.1% BSA in 4°C overnight. Secondary antibody was incubated in 1:500 dilution in 0.1% BSA for 1 hour. Primary antibodies utilized were rabbit polyclonal anti-cTnT (abcam; ab45932) and mouse anti-sarcomeric anti–α-actinin (Abcam; EA-53 ab9465). Secondary antibodies utilized were goat anti-rabbit Alexa Fluor 488 (Abcam; ab150077) and goat anti-mouse Alexa Fluor 594 (Abcam; 150116). Nuclei were stained using DAPI (Thermo Fisher Scientific) at 1:1000 dilution in PBS for 20 minutes. For the haCMs stained using the nonadherent protocol, the cell suspension was gently pipetted onto confocal grade polymer-bottom dishes (ibidi). The cells were visualized using Zeiss Laser Scanning confocal microscope (LSM 710) (META) with 63× oil objective and analyzed on ImageJ (NIH). Sarcomere length was determined by measuring the distance between long axis intensity peaks indicating cellular sarcomeric striations (stained with α-actinin). Ten sarcomere lengths were obtained per cell, examining 12–43 cells.

### Seahorse sample prep, run, and analysis.

We measured OCRs using the Seahorse XFe96 Analyzer. Four days prior to running the assay, the monolayers were dissociated into single cells and replated onto FBN-coated Seahorse XFe96 cell culture microplates at a density of 40,000 cell/80 μL. Media were exchanged every other day until the day of the experiment. The assay was performed in bicarbonate-free RPMI (Agilent Technologies) supplemented with 1% 10 mM glucose, 1% 1 mM pyruvate, and 1% 2 mM glutamine. One day prior to running the Seahorse Assay, the Seahorse XFe96 sensor cartridge was hydrated by submerging the cartridge in Seahorse XF calibrant and incubating the cartridge in a non-CO_2_ 37°C incubator overnight. On the day of the assay, the cells were rinsed with RPMI, then incubated in 100 μL of assay media in a non-CO_2_ 37°C incubator. Effective concentrations of each drug were as follows: 2.5 μM oligomycin, 5 μM FCCP, and 2.5 μM Antimycin A + Rotenone. After running the Seahorse assay, all the data points were normalized to cell number using Presto Blue (Invitrogen).

### Calcium handling imaging.

Fluo-4-AM (Invitrogen) was used to measure calcium transients with the dye dissolved in 2.5% Pluronic F-127 (MilliporeSigma). The dye solution was added to 1 mM Ca^2+^ Tyrode’s solution to a working concentration of 5 μM. Fluo-4-AM in 1 mM Ca^2+^ Tyrode’s solution was added to the cells and incubated in the dark for 20 minutes at room temperature. The cells were washed with indicator-free Tyrode’s solution with 2 mM calcium. Line scans were obtained using the Zeiss LSM 710 confocal equipped with BiG module at 40× objective and analyzed using ImageJ. Fluorescence normalization was based on a baseline background region specific to each cell for corrected minimum and maximum fluorescence values. Temporal fluorescence intensity changes were represented by the calcium transient trace. Calcium transient amplitudes were obtained by integrating fluorescence intensity for the area below peak maxima relative to baseline.

### Whole-cell current-clamp recording.

Patch-clamp measurements were performed as previously described (16). Whole-cell configurations were achieved by using an Axopatch 200B amplifier controlled by pClamp10 software through an Axon Digidata 1440A. The AP was obtained using the current-clamp mode by injecting stimulus current at a frequency of 1 Hz. The current-clamp recordings were recorded in a solution containing 140 mM NaCl, 5.4 mM KCl, 1 mM MgCl_2_, 10 mM glucose, 2.0 mM CaCl_2_, and 10 mM HEPES (pH 7.4 with NaOH). The pipette solution contained 120 mM KCl, 1 mM MgCl_2_, 10 mM HEPES, 2 mM Mg-ATP, and 10 mM EGTA (pH 7.3 with KOH).

### Whole-cell voltage-clamp for I_Ks_.

For I_Ks_ recordings, the external solution contained 140 mM NaCl, 4 mM KCl, 1.8 mM CaCl2, 1.2 mM MgCl_2_, 10 mM glucose, 10 mM HEPES, and 0.01 mM nifedipine, adjusted to pH 7.4 with NaOH. I_Ks_ recordings were isolated as 1 μM HMR-1556–sensitive current. An intracellular solution contained 100 mM potassium aspartate, 20 mM KCl, 2 mM MgCl_2_, 5 mM Mg-ATP, 5 mM EGTA, 10 mM HEPES, and Amphotericin-B 0.44 mM, adjusted to 7.2 with KOH. I_Ks_ currents were elicited by using 3-second voltage-clamp steps to test potentials of −60 to +60 mV from holding potential of –40 mV and with 20 mV increments.

### Optical voltage mapping.

Cells were washed 5 times with indicator-free Tyrode’s solution. After reconstituting the 2 mM VF2.1Cl dye to 1 mM in 10% Pluronic F-127, then diluting the dye to 100 nM in Tyrode’s solution, the VF2.1Cl dye (MilliporeSigma) was added to each well for 50 minutes in a 37°C 5% CO_2_ incubator. The cells were then washed 5 times with indicator-free Tyrode’s solution and returned to the 37°C 5% CO_2_ incubator for 10 minutes to recover. The dye was excited at 514 nm wavelength, and time series images were acquired at an acquisition frequency of 45 Hz for 40 seconds in Epi-fluorescence mode using Zeiss Laser TIRF Microscope fitted with a Hamamatsu ORCA-Flash 4.0 V3 digital CMOS camera C13440-20CU. HMR-1556 (Tocris) was added at a concentration of 1 μM 30 minutes prior to image acquisition and incubated in a 37°C 5% CO_2_ incubator. APD_90_ was calculated as described previously (55).

### RNA isolation, cDNA synthesis, and RT-PCR.

Total RNA was extracted using TRIzol (Invitrogen) phenol-chloroform extraction and reverse-transcribed using SuperScript III Reverse Transcriptase (Thermo Fisher Scientific) according to manufacturer-recommended procedures. RT-PCR was performed with FastStart Universal SYBR Green Master Mix or TaqMan Fast Advanced Master Mix (see Supplemental Table 2 for all primers and probes used). Expression of mRNA was normalized to GAPDH, and data were analyzed with the ΔΔCt method.

### RNA-Seq.

RNA quality and quantity were assessed using the Agilent Bioanalyzer. Strand-specific RNA-Seq libraries were prepared using a TruSEQ mRNA-Seq library protocol (Illumina provided). Library quality and quantity were assessed using the Agilent Bioanalyzer, and libraries were sequenced using an Illumina NovaSEQ6000 (Illumina-provided reagents and protocols). Data were demultiplexed using Illumina provided bcl2fastq software.

### TF network analysis.

Upstream regulator analysis was performed using the core analysis function of IPA for up- and downregulated DEGs (FDR < 0.05). Differentially expressed TF target genes for significantly enriched (Benjamini-Hochberg correction, *P* < 0.05) TFs were obtained. These genes for each TF were intersected with genes from GO terms heart contraction (GO:0060047), myofibril assembly (GO:0030239), and oxidative phosphorylation (GO:0006119), and enrichment statistics of this intersection were computed using Fisher’s exact test. Network plots of differentially expressed target genes for select TFs within select pathways were visualized using the graph package in R (https://CRAN.R-project.org/package=ggraph), with a stress layout.

### Protein isolation and Western blots.

Western blots were performed as previously described (16, 30). Cells on 6-well plates were washed with ice-cold DPBS without Ca^2+^ and Mg^2+^, after which 250 μL of 1× RIPA with protease and phosphatase inhibitors was added per well. Lysate concentrations were quantified using BCA assay (Thermo Fisher Scientific) and diluted with 4× Laemmli buffer with 10% 2-mercaptoethanol. Mitochondrial Isolation Kit for cultured cells (Abcam) was used to probe mitochondria-specific proteins. Per sample, 25 μg of protein was then size-fractioned on an SDS-PAGE gel, and resolved gels were electro-transferred on 0.2 μm PVDF membranes. Membranes were blocked with 5% BSA for 1 hour, then probed with corresponding antibodies of target proteins (Supplemental Table 3). The blots were developed using either anti-rabbit HRP or anti-mouse HRP and scanned on C280 imaging systems (Azure Biosystems). Protein signal densities were determined using ImageJ and normalized to corresponding β-actin signal densities.

### Accession numbers.

The RNA-Seq data reported in this manuscript were deposited into the National Center for Biotechnology Information’s Gene Expression Omnibus database with the accession number GSE193856.

### Statistics.

Unless otherwise noted, data were presented as mean ± SD. For data with normal distribution, nonparametric unpaired and 2-tailed Mann-Whitney *U* test was used to determine statistical significance between 2 groups and either 1-way or 2-way ANOVA for multiple groups with post hoc Bonferroni’s corrections. Significance was notated as *P < 0.05, **P < 0.01, ***P < 0.001, ****P < 0.0001, with *P* < 0.05 being considered significant.

### Study approval.

We used the UIC Institutional Review Board–approved protocol to enroll participants following receipt of informed written consent.

## Author contributions

OTL, HC, SRK, and DD designed the experiments. OTL performed iPSC culture and iPSC-aCM differentiation, patient recruitment and haCM isolation, IF, RNA isolation and sample prep for RT-PCR and RNA-Seq, RT-PCR, Western blots, calcium transients, optical voltage mapping, and data analysis and wrote the manuscript. OTL and SGO performed Seahorse Analyzer experiments. HC generated, reprogrammed, and analyzed the iPSC and CRISPR/Cas9-GC lines. LH performed EP whole-cell current-clamp recordings and analyzed the data. GEB engineered coverslips required for all EP experiments. XW and JR performed and interpreted the RNA-Seq analysis. YDH designed and fabricated electrical stimulator setup used for preliminary experiments. MAP performed EP whole-cell voltage-clamp recordings and analyzed the data. AS assisted with experimental analysis. MMC and JR performed upstream TF regulator analysis. BC recruited patients for L3, *NPPA*-WT, and *NPPA*-S64R. KA, MM, and LER provided HAT and whole blood for PBMC extraction. DD, SRK, and JR provided critical revisions of the manuscript. DD, JR, and SRK supervised experiments and provided funding support. All authors provided critical feedback and contributed to the final manuscript.

## Supplementary Material

Supplemental data

## Version 1 04/08/2022

Electronic publication
