# Supplementary material for: Mutant ANP induces mitochondrial and ion channel remodeling in a human iPSC–derived atrial fibrillation model
Source: JCI Insight. 2022 Apr 8;7(7):e155640. doi: 10.1172/jci.insight.155640 (PMC9057627; doi:10.1172/jci.insight.155640)
Supplement: Supplemental data [file jciinsight-7-155640-s008.pdf]

Cell Line	Sex	Age at Recruitment	Ethnicity	Surgical Procedure	Human Atrial Biopsy
**P1**	M	74	African American	Coronary Artery Bypass Graft	Yes
**P2**	M	59	African American	Coronary Artery Bypass Graft	Yes
**L3**	F	48	Caucasian	--	No
***NPPA*-WT**	M	46	Caucasian	--	No
***NPPA*-S64R**	M	42	Caucasian	--	No

Table S1: Clinical characteristics of patients from whom induced pluripotent stem cells (iPSC) lines were derived.

Supplementary Figures

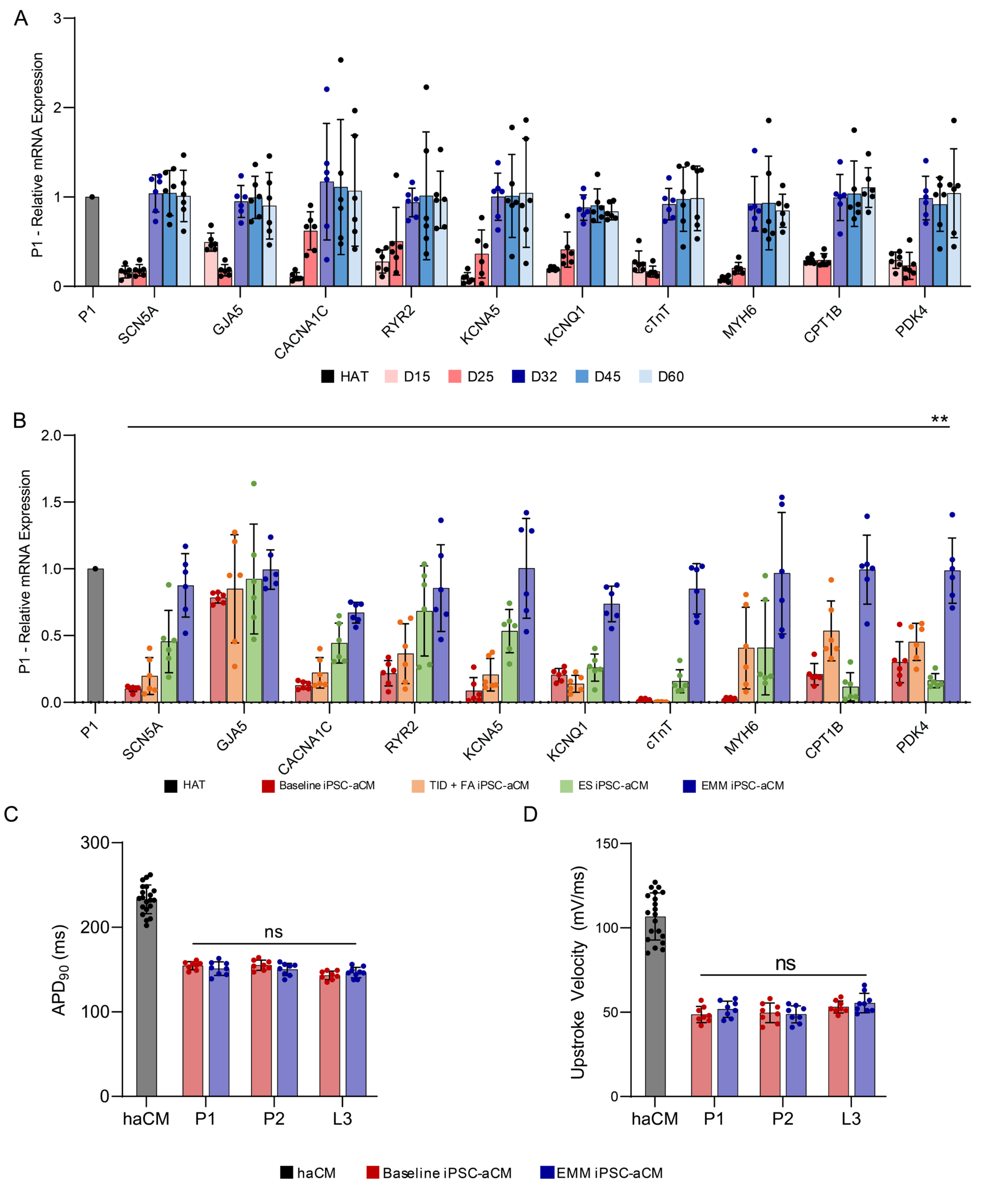

Figure S1. Optimization of maturation protocol length and stimulation parameters: A) Molecular characterization of select ion channels, gap junctions, structural genes, and fatty acid (FA) metabolism genes

shows that by day 32, iPSC-derived atrial cardiomyocytes (aCMs) undergoing triiodothyronine (T), insulin-like growth factor-3 (I) and dexamethasone (D; collectively TID) + FA + electrical stimulation (ES) reach an expression level equivalent to that of human atrial tissue (HAT) from the same patient. This expression level does not improve past day 32 (examining time points Day 45 and Day 60), establishing that with this method of maturation, 32 days is sufficient to achieve the optimal level of maturation. **B**) Molecular characterization of select ion channels, gap junctions, structural genes, and FA metabolism genes shows that while applying TID+FA only and ES only to the iPSC-aCMs does display some improvement compared to baseline iPSC-aCMs, optimal level of maturation established by HAT from the same patient is only synergistically achieved with EMM conditioning. EMM does not improve APD_{90} (**C**) or upstroke velocity (**D**) despite molecular improvement in relevant ion channels. *P<0.05, **P<0.01, ***P<0.001, ****P<0.0001 (2-way ANOVA with post-hoc Bonferroni corrections).

Supplementary Figures

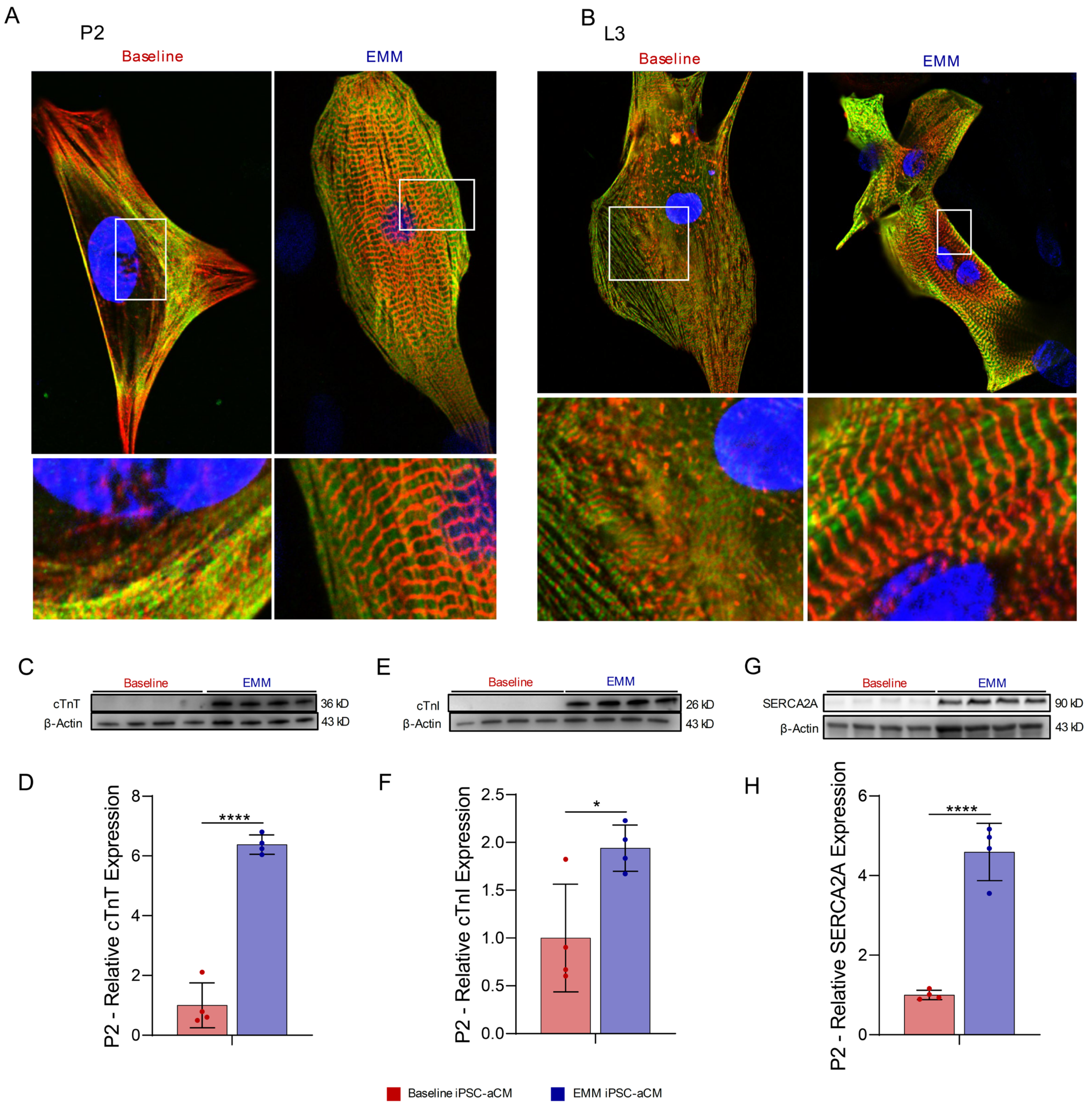

α-actinin show that in two additional independently derived cells lines P2 (**A**) and L3 (**B**), EMM results in a more elongated cell morphology and sarcomeric organization in both the periphery and perinuclear region compared to baseline iPSC-aCMs. In an additional cell line (L3), EMM iPSC-aCMs also demonstrated

increased protein expression of cTnT (**C,D**), cTnI (**E,F**), and SERCA2A (**G,H**). *P<0.05, **P<0.01, ***P<0.001, ****P<0.0001 (nonparametric Mann-Whitney *U* test).

Supplementary Figures

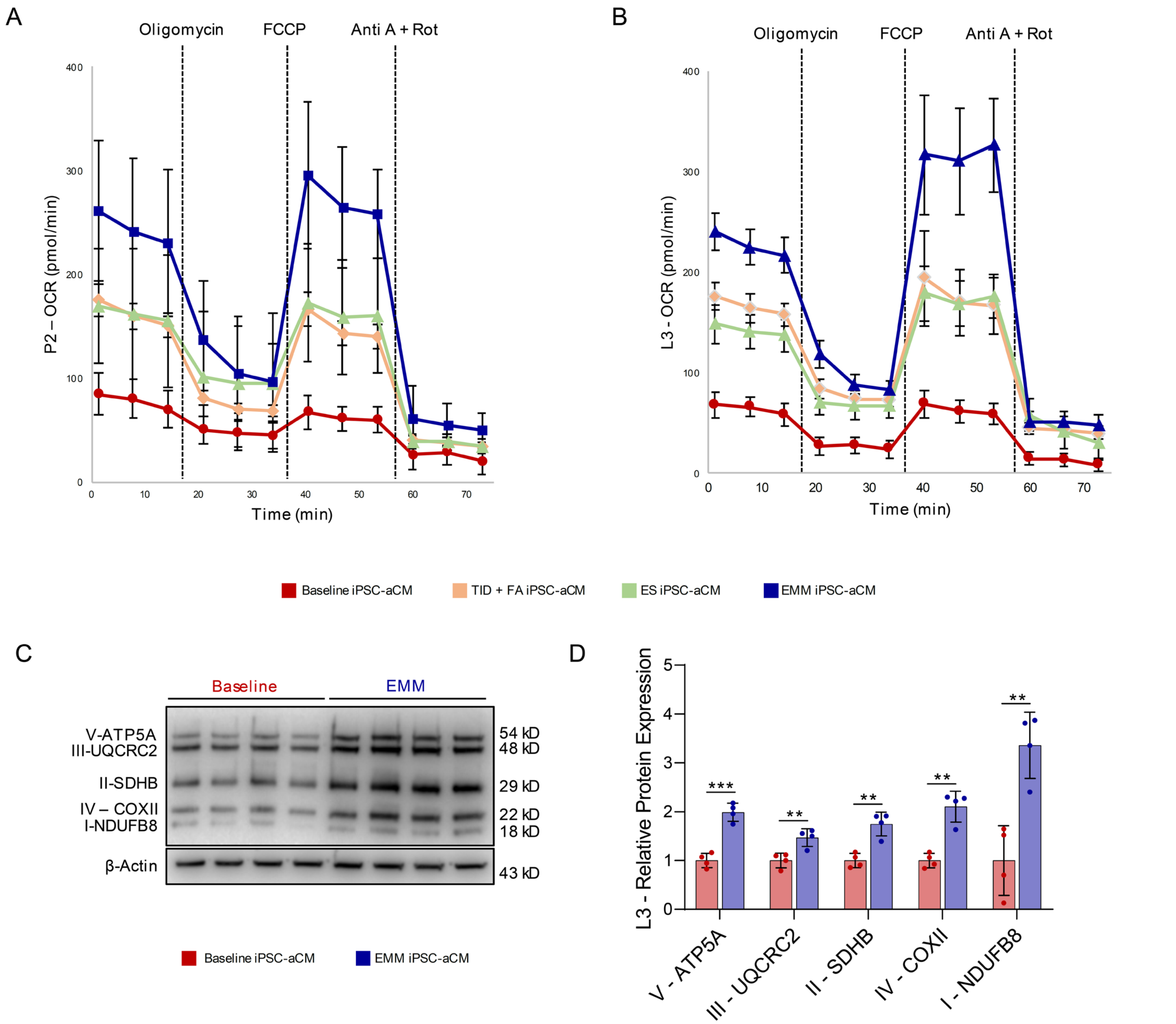

Figure S3. Metabolic maturity is achieved in two additional cell lines: a. Real-time oxygen consumption rate (OCR) measurements in two additional cell lines of baseline, TID+FA only, ES only, and EMM iPSC-aCMs by Seahorse Xfe96 showed that EMM iPSC-aCMs exhibited the highest respiration rate under basal conditions and after mitochondrial uncoupling for both P2 (**A**) and L3 (**B**). Western blots of mitochondrial oxidative phosphorylation (**C**) genes in L3 shows that EMM significantly upregulates expression of each of the five mitochondrial complexes involved in mitochondrial oxidative phosphorylation (**D**). (n = 4 biological replicates). *P<0.05, **P<0.01, ***P<0.001, ****P<0.0001 (nonparametric Mann-Whitney *U* test).

Supplementary Figures

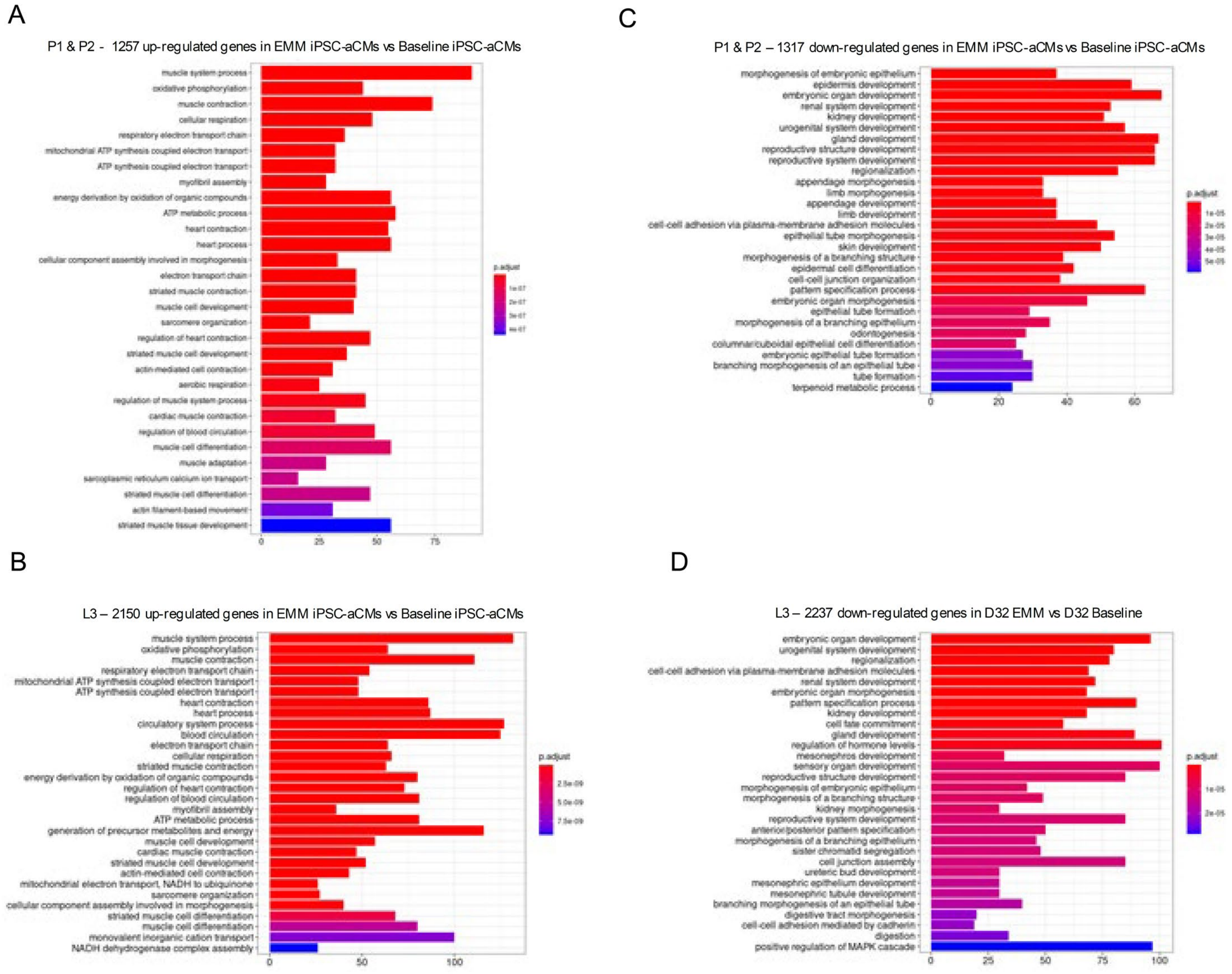

Figure S4: Gene ontology (GO) enrichment analysis of EMM iPSC-aCMs compared to baseline iPSC-aCMs: A-B) The top 30 GO pathways that the upregulated differentially expressed genes (DEGs) in EMM iPSC-aCMs compared to baseline iPSC-aCMs in P1 and P2 (**A**), and L3 (**B**) mapped primarily to included key maturation GO pathways such as heart contraction, cardiac muscle contraction, sarcomere organization, myofibril assembly, oxidative phosphorylation, and mitochondrial metabolism, showing that EMM iPSC-aCMs achieved a much higher level of maturity compared to baseline iPSC-aCMs. **C-D**) The top 30 GO pathways that the downregulated DEGs in EMM iPSC-aCMs compared to baseline iPSC-aCMs in P1, P2, and L3 mapped included embryonic development, cellular proliferation, and extracardiac development.

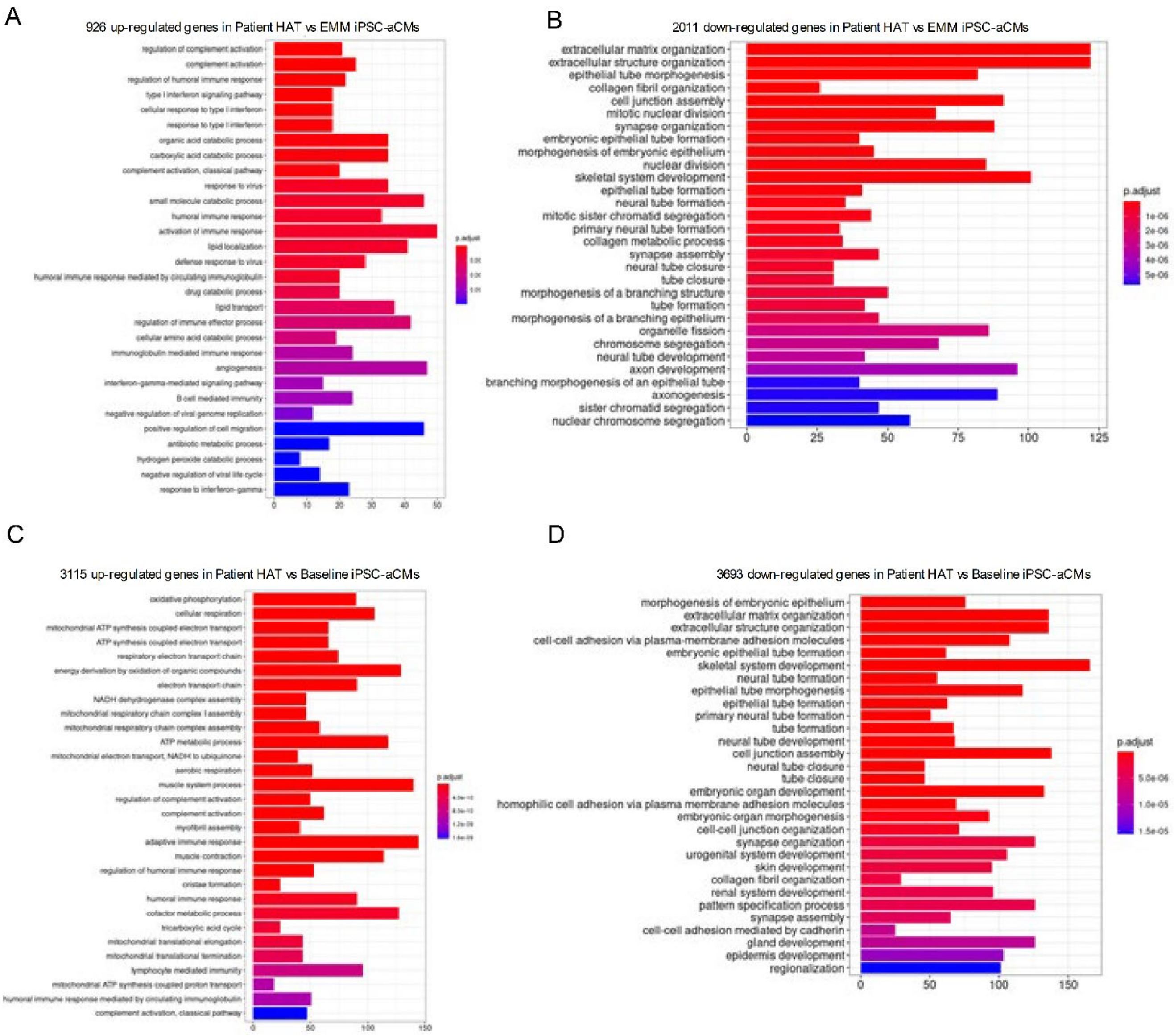

Figure S5: GO enrichment analysis of patient derived iPSC-aCMs compared to HAT from the same patient: A) The top 30 GO pathways that the 926 upregulated DEGs in HAT compared to EMM iPSC-aCMs from the same patient mapped to primarily related to immune response, among others. The absence of key maturation GO pathways such as heart contraction, sarcomeric and myofibril assembly, and mitochondrial metabolism and oxidative phosphorylation show that these pathways are no longer in the top 30 upregulated pathways comparing HAT with EMM iPSC-aCMs. These key GO pathways were also no longer different (q>0.05) when comparing HAT with EMM iPSC-aCMs. **B**) The top 30 GO pathways that the 2011 downregulated DEGs in HAT compared to EMM iPSC-aCMs from the same patient mapped to included cellular proliferation, embryonic development, and extracardiac development. **C**) The top 30 GO pathways that

the 3115 upregulated DEGs in HAT compared to baseline iPSC-aCMs mapped to show that HAT differentially expressed much higher levels of genes related to key maturation GO pathways. **D**) The top 30 GO pathways that the 2693 downregulated DEGs in HAT compared to baseline iPSC-aCMs mapped included extracardiac development, embryonic development, and cellular proliferation.

Supplementary Figures

A

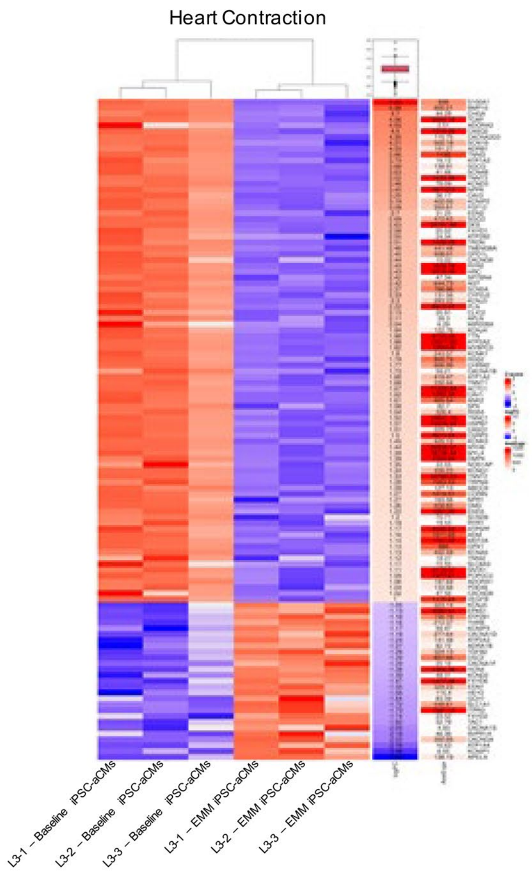

B

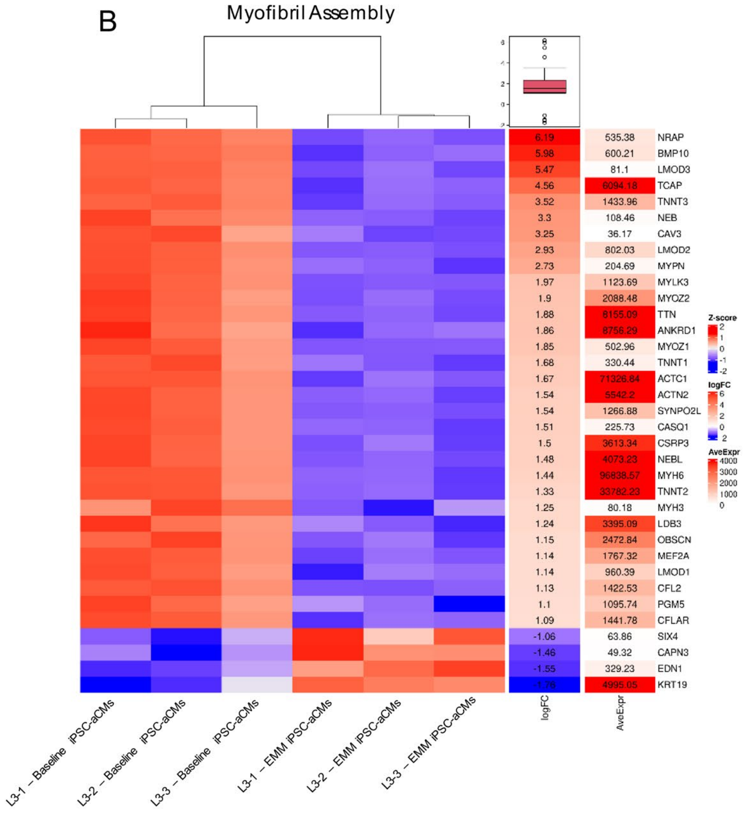

C

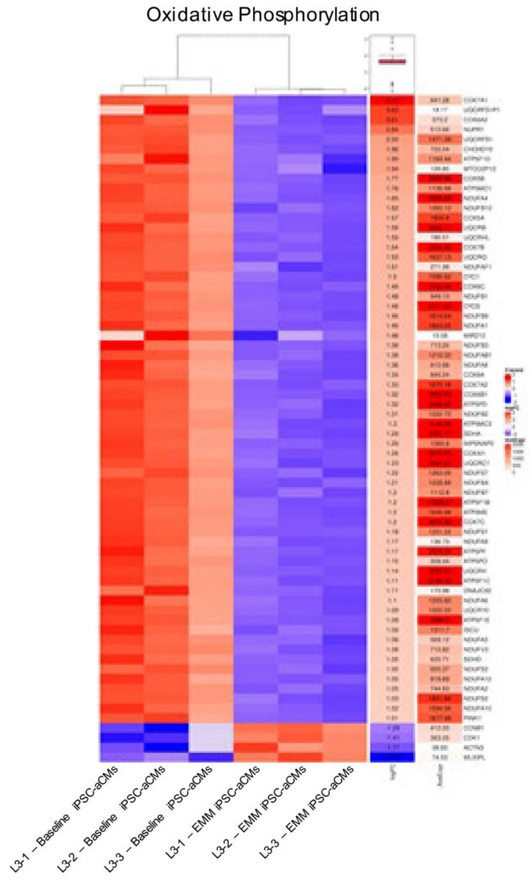

D

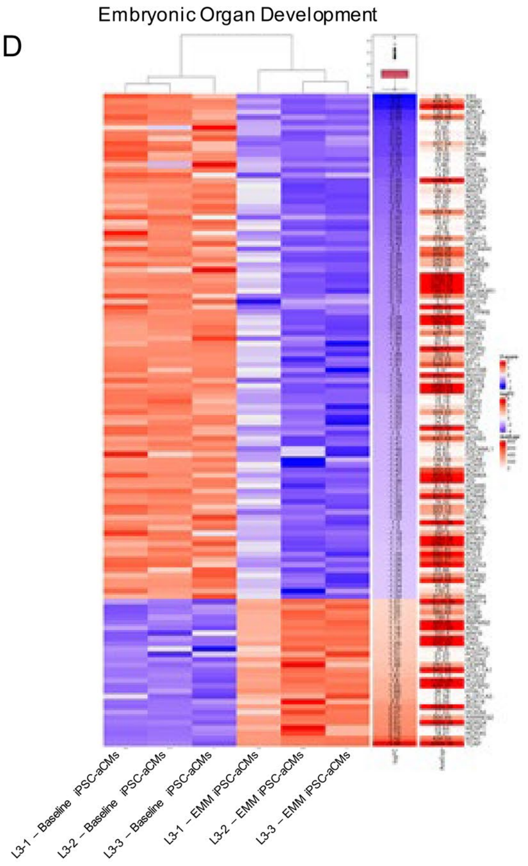

Figure S6: Heatmaps of DEGs associated with targeted GO pathways: Heatmaps of upregulated and downregulated DEGs mapping to target GO pathways Heart Contraction (**A**), Myofibril Assembly (**B**), Oxidative Phosphorylation (**C**), and Embryonic Organ Development (**D**) show that genes related to heart contraction, myofibril assembly, and oxidative phosphorylation were significantly upregulated in EMM iPSC-aCMs compared to baseline iPSC-aCMs ($q<0.05$). Genes related to embryonic organ development were also significantly downregulated in EMM iPSC-aCMs compared to baseline iPSC-aCMs ($q<0.05$). Comparison to HAT was not performed in L3 since this patient was not undergoing cardiac surgery at the time of recruitment.

Supplementary Figures

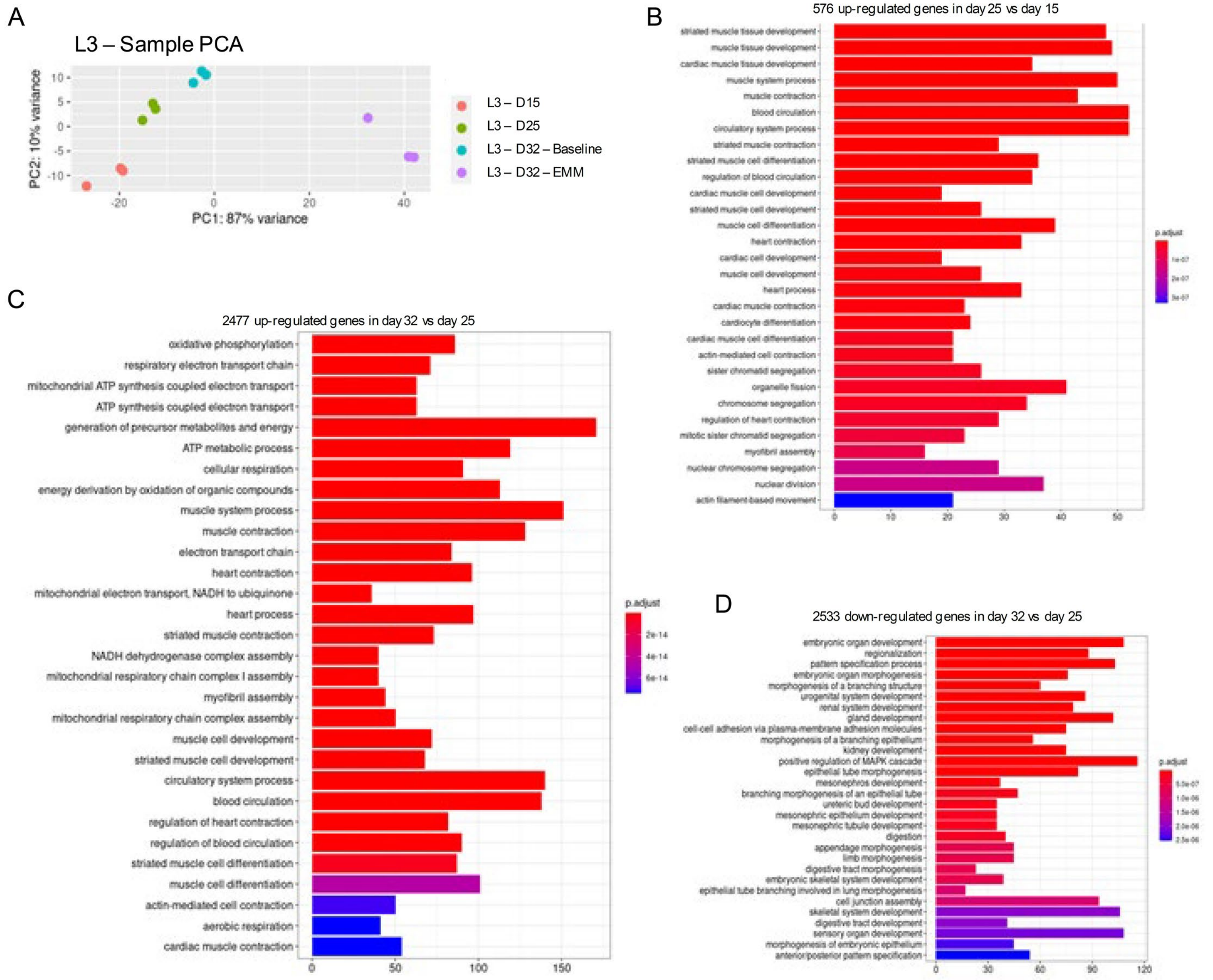

Figure S7: Time series gene ontology enrichment analysis of maturation of EMM iPSC-aCM: A) Sample principal component analysis (PCA) plot of L3 at day 15 (fetal), day 25 (midway through maturation), and day 32 (completed maturation). Three samples from each time point in independent experiments were sequenced. Samples clustered well with minimal variation within each experimental group. **B**) The top 30 GO pathways that the 576 upregulated DEGs comparing day 25 to day 15 mapped to primarily included cardiac and muscle cell and process development as well as cellular proliferation. There were no significant GO pathways downregulated comparing day 25 to day 15. **C**) The top 30 GO pathways that the 2477 upregulated DEGs comparing day 32 to day 25 mapped to included oxidative phosphorylation and mature metabolic processes, as well as heart and muscle contraction, and myofibril assembly. **D**) The top 30 GO pathways that the 2533

downregulated DEGs comparing day 32 to day 25 mapped to included embryonic development and extracardiac development.

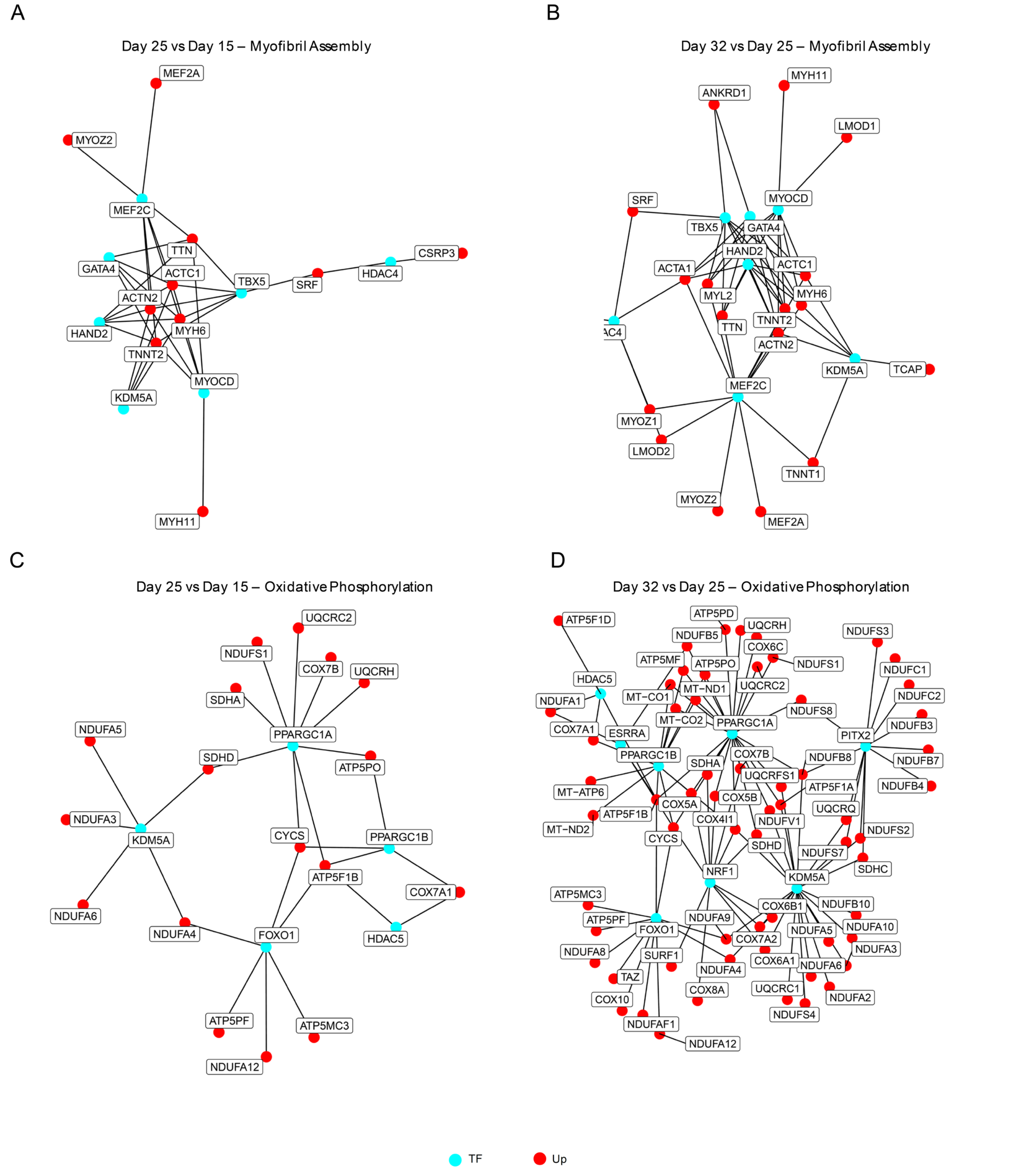

Figure S8: Transcription factor (TF) network analysis of the time series: The same 7 regulatory TFs were targeted in Myofibril Assembly to examine the increased target enrichment of the same TFs, comparing day 25

vs day 15 (**A**) with day 32 vs day 25 (**B**). Target regulatory TFs in Oxidative Phosphorylation were chosen to highlight to higher number of enriched isoforms of each complex involved in mitochondrial oxidative phosphorylation, comparing day 25 vs day 15 (**C**) with day 32 vs day 25 (**D**).

Supplementary Figures

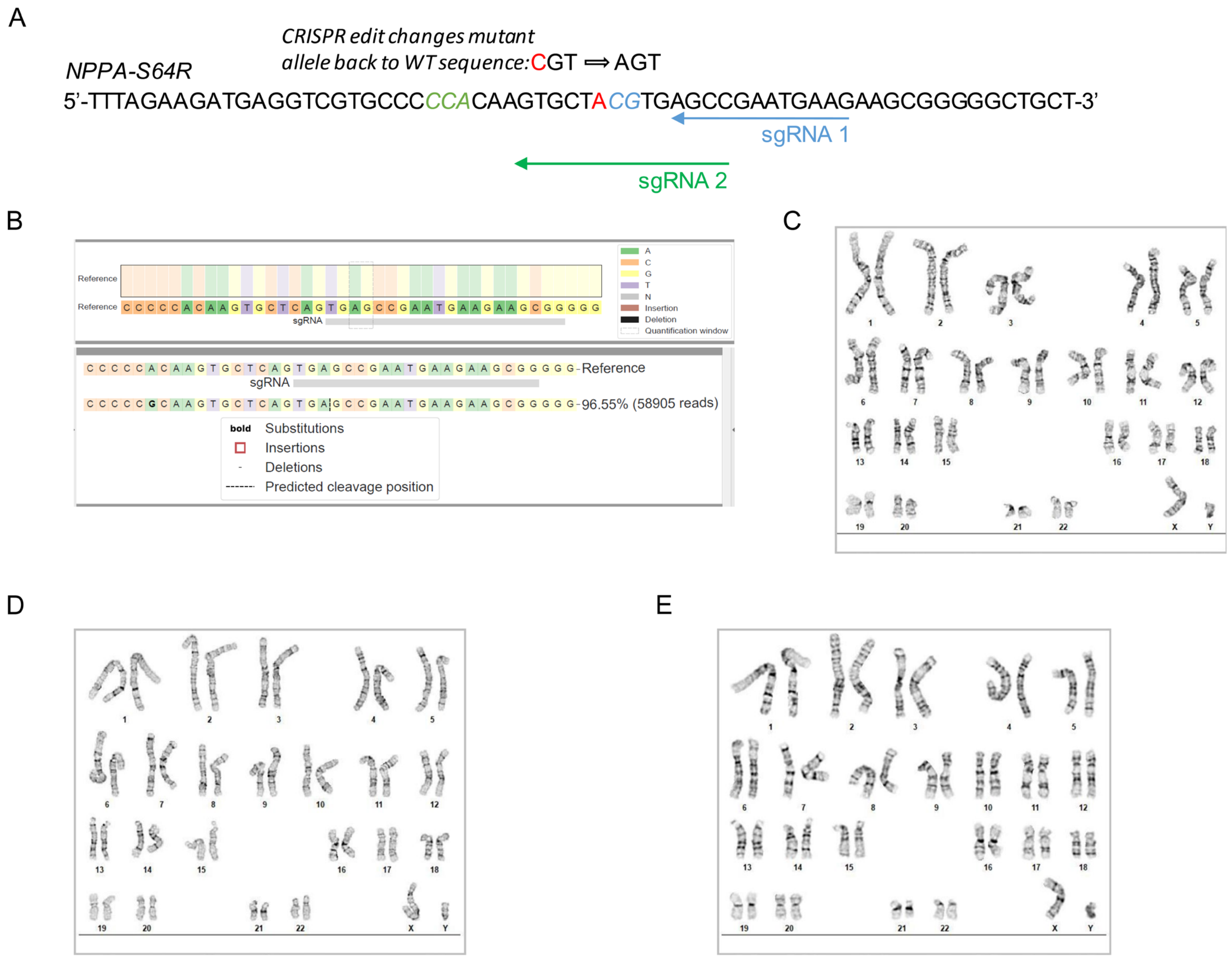

Figure S9: CRISPR-Cas9-mediated genome correction (GC) of the atrial fibrillation (AF) iPSC line carrying the *NPPA*-S64R mutation: A) *NPPA* CRISPR (guide) gRNA design for reversion of *NPPA-S64R* mutation. Genomic *NPPA* sequence is annotated for A to C mutation (red) and the location of protospacer adjacent motive (PAM) sequences (blue or green) for the two single guide (sg)RNA (arrows, blue or green) that we will independently use to generate the iPSC line with the repaired *NPPA* gene. **B**) Next generation sequencing (NGS) results summary showing successful positive gene editing. (**C-E**) Karyotyping analysis for *NPPA*-S64R-GC (**C**), P1 (control non-AF) (**D**), and P2 (control non-AF) (**E**) iPSCs demonstrate normal karyotype.

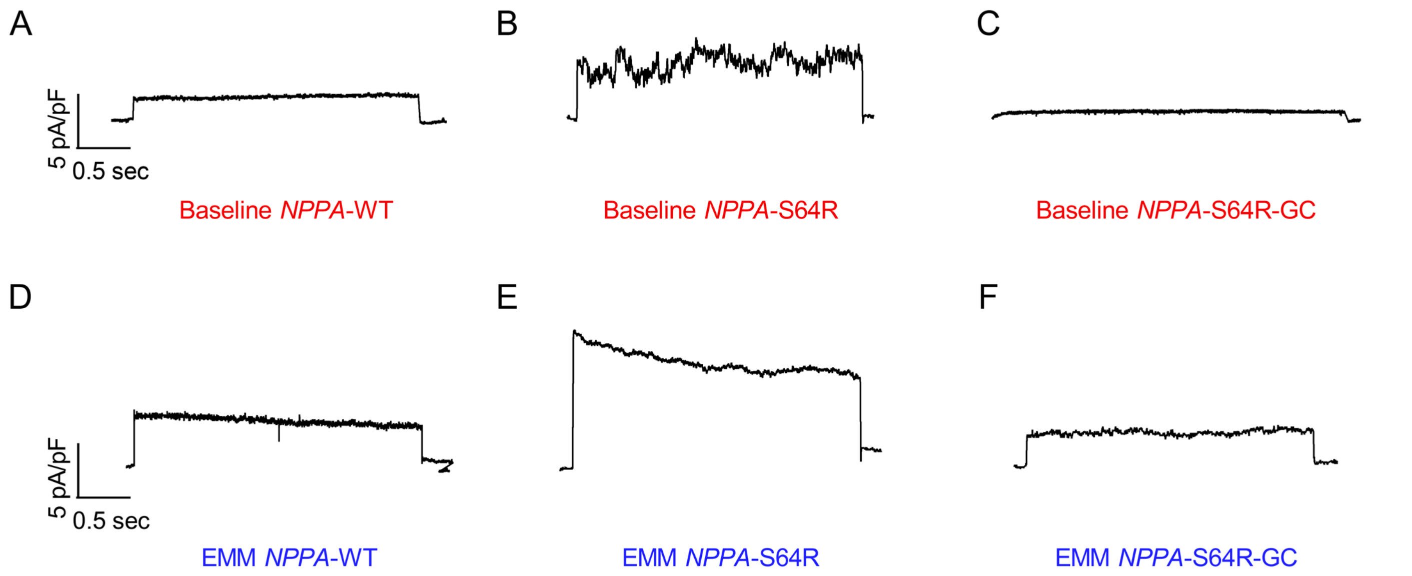

Figure S10: Current tracings for the slow delayed rectifier potassium current (I_{Ks}): (**A-C**) Representative I_{Ks} recordings for baseline *NPPA*-WT (**A**), *NPPA*-S64R (**B**), and *NPPA*-S64R-GC (**C**) iPSC-aCMs. (**D-F**) Representative I_{Ks} recordings for EMM *NPPA*-WT (**D**), *NPPA*-S64R (**E**) , and *NPPA*-S64R-GC (**F**) iPSC-aCMs.

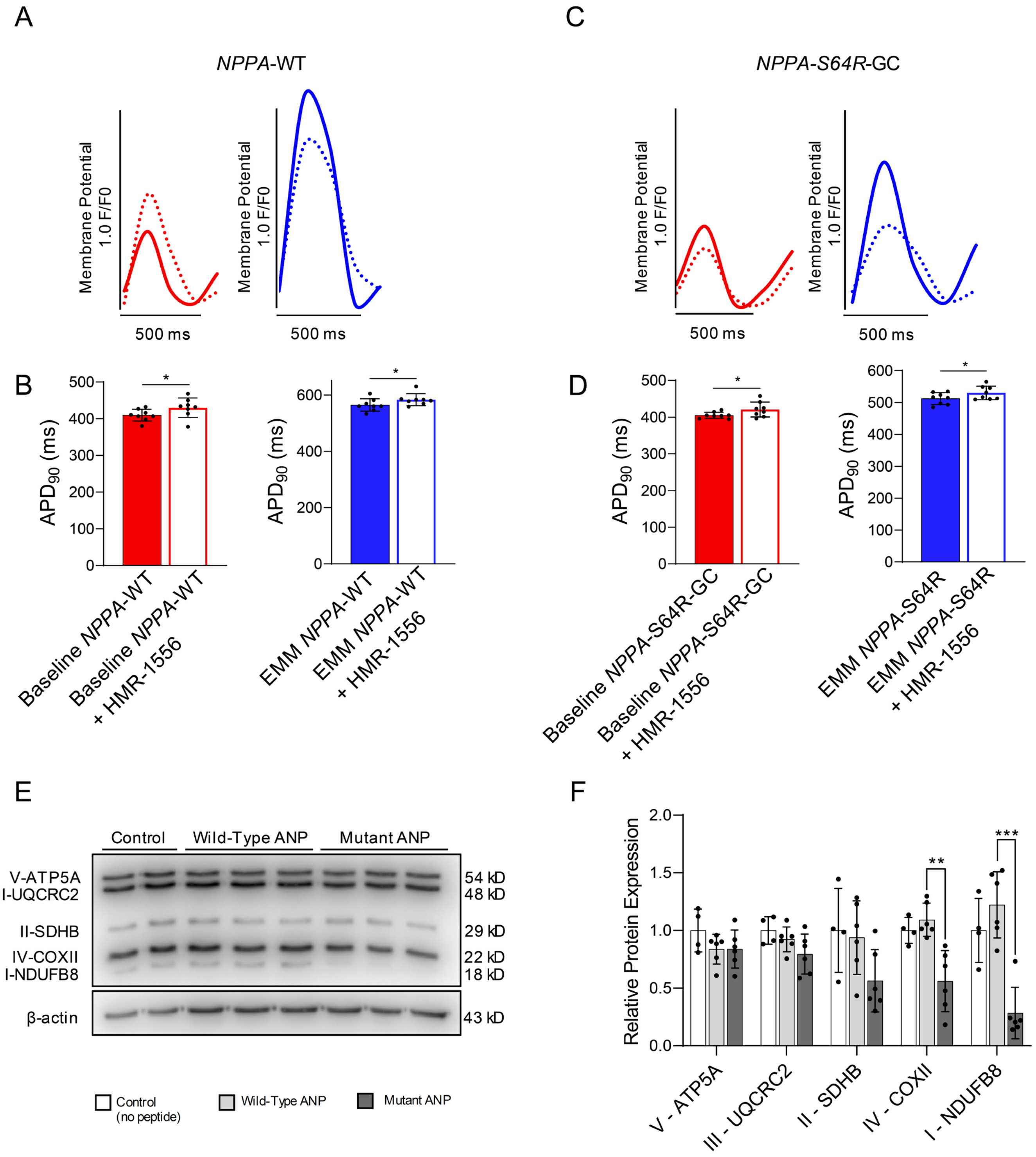

Figure S11: Electrophysiological (EP) and metabolic substrate underlying *NPPA*-S64R mutation. A-D) APD_{90} quantification of *NPPA*-WT iPSC-aCMs (**A-B**) and *NPPA*-S64R-GC (**C-D**), comparing baseline iPSC-aCMs (red) with EMM iPSC-aCMs (blue). APD_{90} prolongation with administration of I_{Ks} blocker in EMM *NPPA*-

S64R iPSC-aCMs is much more striking when compared to that of baseline *NPPA*-S64R iPSC-aCMs; n = 2 batches, n = 4 cells per batch (nonparametric Mann-Whitney *U* test). **E-F**) Infusion of wild-type (WT) ANP and mutant ANP produced due to *NPPA*-S64R mutation demonstrated the immediate modulation of complex I protein expression due to the mutation and subsequent mutant peptide (2-way ANOVA with Bonferroni correction); ns = no significant difference, *P<0.05, **P<0.01, ***P<0.001, ****P<0.0001

Gene	Primer/Probe	Method
GAPDH	Forward 5'-AGCCACATCGCTCAGACACC-3'<br>Reverse 5'-GTACTCAGCGCCAGCATCG-3'	SYBR
SCN5A	Forward 5'-GAAGAAGCTGGGCTCCAAGA-3'<br>Reverse 5'-CATCGAAGGCCTGCTTGGTC-3'	SYBR
GJA1	Forward 5'-GAAGAAGCTGGGCTCCAAGA-3'<br>Reverse 5'-CATCGAAGGCCTGCTTGGTC-3'	SYBR
GJA5	Forward 5'-GAAGAAGCTGGGCTCCAAGA-3'<br>Reverse 5'-CATCGAAGGCCTGCTTGGTC-3'	SYBR
CACNA1C	Forward 5'-CCAACCTCATCCTCTTCTTCA-3'<br>Reverse 5'-ACATAGTCTGCATTGCCTAGGAT-3'	SYBR
RYR2	Forward 5'-AAGGCGAGGATGAGATCCAG-3'<br>Reverse 5'-TTCTTTGTGGATGGTCGCCG-3'	SYBR
SERCA2	Forward 5'-GGTGCTGAAAATCTCCTTGC-3'<br>Reverse 5'-ATCAGTCATGCACAGGGTTG-3'	SYBR
KCNA5	Hs00969279_s1	TaqMan
KCNQ1	Hs00923522_m1	TaqMan
KCNE1	Hs00899753_m1	TaqMan
KCND3	Forward 5'-CTTCGCGGAAGGGTTTGC-3'<br>Reverse 5'-GTGAAGATCATGACGCACGC-3'	SYBR
KCNJ3	Forward 5'-TGAGGGACGGAAAACTCACG-3'<br>Reverse 5'-GACAAGTCATCCTTTGAGCAGC-3'	SYBR
TNNT2	Forward 5'-GGCAGCGGAAGAGGATGCTGAA-3'<br>Reverse 5'-GAGGCACCAAGTTGGGCATGAACGA-3'	SYBR
MYH6	Forward 5'-CCAGAGCTTGCTGAAGGACA-3'<br>Reverse 5'-TTGGCAAGAGTGAGGTTCCC-3'	SYBR
CPT1B	Forward 5'-ACTCCTGGAAGAAACGCCTG-3'<br>Reverse 5'-CACAGACTCTAGGTAAGCCCAG-3'	SYBR
PDK4	Forward 5'-CCTGTGAGACTCGCCAACAT-3'<br>Reverse 5'-GTTCAACTGTTGCCCGCATT-3'	SYBR
PPARGC1A	Forward 5'-CCCCATGGATGAAGGGTACTT-3'<br>Reverse 5'-GGGGAGGTCTCATCCATTGC-3'	SYBR
ATP5A	Forward 5'-GTATTGCCCGCGTACATGG-3'<br>Reverse 5'-AGGACATACCCTTTAAGCCTGA-3'	SYBR
UQCRC2	Forward 5'-TTCAGCAATTTAGGAACCACCC-3'<br>Reverse 5'-GGTCACACTTAATTTGCCACCAA-3'	SYBR
SDHB	Forward 5'-ACAGCTCCCCGTATCAAGAAA-3'<br>Reverse 5'-GCATGATCTTCGGAAGGTCAA-3'	SYBR
MT-CO2	Forward 5'-CTGATCTGCGGCTACAATTCTG-3'<br>Reverse 5'-CCCGGAAGAGGACTTGCTT-3'	SYBR
NDUFB8	Forward 5'-CCGCCAAGAAGTATAATATGCGT-3'<br>Reverse 5'-TATCCACACGGTTGTTGT-3'	SYBR

Table S2: Primers used for RT-PCR to determine relative expression levels in iPSC-aCMs and HAT.

Antibody	Antibody vendor	Dilution	Experiment
anti-cTnT polyclonal (rabbit)	proteintech (15513-1-AP)	1:500	Western Blot
anti-cTnT polyclonal (rabbit)	abcam (ab45932)	1:200	Immunofluorescence
Anti-Sarcomeric α-actinin (mouse)	abcam (EA-53 ab9465)	1:200	Immunofluorescence
anti-cTnI polyclonal (rabbit)	abcam (ab47003)	1:500	Western Blot
anti-SERCA2 polyclonal (rabbit)	alomone (APC-012	1:300	Western Blot
Total OXPHOS Human WB Antibody Cocktail (mouse)	abcam (ab110411)	1:1000	Western Blot
anti-KCNQ1 polyclonal (rabbit)	alomone (APC-022)	1:500	Western Blot
anti-rabbit HRP	Cell Signaling Technology (7074)	1:1500	Western Blot
anti-mouse HRP	Cell Signaling Technology (7076)	1:1500	Western Blot
Goat anti-rabbit Alexa Fluor-488	abcam (ab150077)	1:1000	Immunofluorescence
Goat anti-mouse Alexa Fluor-594	abcam (ab150116)	1:1000	Immunofluorescence
DAPI	Thermo Scientific (62248)	1:1000	Immunofluorescence

Table S3: Antibodies used in Western blots and immunofluorescence to probe protein expression levels and localization.
